# Silicon Oxycarbide (SiOC)-Supported Ionic Liquids: Heterogeneous Catalysts for Cyclic Carbonate Formation

**DOI:** 10.1021/acssuschemeng.3c05569

**Published:** 2024-01-18

**Authors:** Philipp Mikšovsky, Katharina Rauchenwald, Shaghayegh Naghdi, Hannah Rabl, Dominik Eder, Thomas Konegger, Katharina Bica-Schröder

**Affiliations:** † Institute of Applied Synthetic Chemistry, TU Wien, Getreidemarkt 9, 1060 Vienna, Austria; ‡ Institute of Chemical Technologies and Analytics, TU Wien, Getreidemarkt 9, 1060 Vienna, Austria; § Institute of Materials Chemistry, TU Wien, Getreidemarkt 9, 1060 Vienna, Austria

**Keywords:** carbon dioxide valorization, bio-based cyclic carbonate, continuous flow, supercritical carbon dioxide, freeze-casting, photopolymerization-assisted solidification templating, polymer-derived ceramics

## Abstract

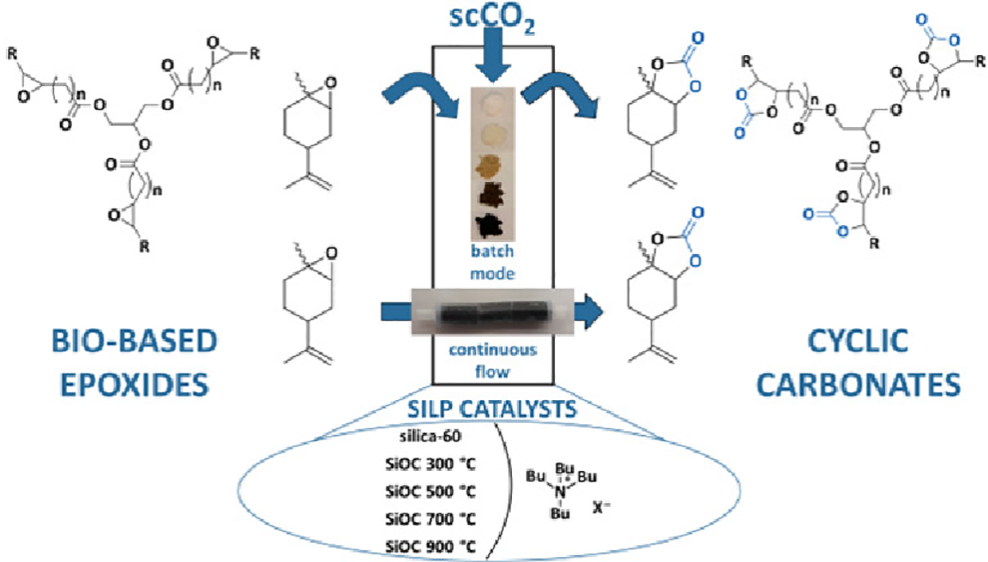

Silicon oxycarbides (SiOCs) impregnated with tetrabutylammonium halides (TBAX) were investigated as an alternative to silica-based supported ionic liquid phases for the production of bio-based cyclic carbonates derived from limonene and linseed oil. The support materials and the supported ionic liquid phases (SILPs) were characterized via Fourier transform infrared spectroscopy, thermogravimetric analysis, nitrogen adsorption, X-ray photoelectron spectroscopy, microscopy, and solvent adsorption. The silicon oxycarbide supports were pyrolyzed at 300–900 °C prior to being coated with different tetrabutylammonium halides and further used as heterogeneous catalysts for the formation of cyclic carbonates in batch mode. Excellent selectivities of 97–100% and yields of 53–62% were obtained with tetrabutylammonium chloride supported on the silicon oxycarbides. For comparison, the catalytic performance of commonly employed silica-supported ionic liquids was investigated under the same conditions. The silica-supported species triggered the formation of a diol as a byproduct, leading to a lower selectivity of 87% and a lower yield of 48%. Ultimately, macroporous monolithic SiOC-SILPs with suitable permeability characteristics (*k*_1_ = 10^–11^ m^2^) were produced via photopolymerization-assisted solidification templating and applied for the selective and continuous production of limonene carbonate with supercritical carbon dioxide as the reagent and sole solvent. Constant product output over 48 h without concurrent catalyst leaching was achieved.

## Abstract 2

Silicon oxycarbide-supported ionic liquids were investigated for the formation of bio-based cyclic carbonates in batch mode and continuous flow using supercritical carbon dioxide.

## Introduction

The concept of supported ionic liquid phases (SILPs) describes materials where a thin layer of ionic liquid remains confined on a porous solid support, resulting in composite materials that possess unique properties.^1^ The combination of the liquid-like behavior of ionic liquids with the structural integrity of solid supports lead to numerous applications that have emerged over the past years, covering catalysis,^2−10^ gas purification, and storage,^11−14^ as well as metal recovery.^15−17^ Especially in catalysis, SILPs combine the properties of ionic liquids with the advantages of heterogeneous systems, such as easy catalyst separation and improved mass transfer. Furthermore, the application of SILPs circumvents the use of bulk quantities of ionic liquids, which is not feasible for their application on an industrial scale.^18−20^

SILPs have also been successfully employed for the immobilization of catalysts in combination with supercritical carbon dioxide (scCO_2_, *T*_c_: 31.0 °C, p_c_: 7.38 MPa),^21^ as scCO_2_ is highly soluble in ionic liquids but ionic liquids cannot dissolve in scCO_2_. This behavior provides ideal conditions for the immobilization of catalysts, where scCO_2_ serves either as the solvent^22−25^ or as the reagent and solvent at the same time.^26−29^ Thus, scCO_2_ can act simultaneously as a C1 building block, for example, in the formation of cyclic carbonates from epoxides, and as the solvent. The aims are to increase the catalytic activity,^30^ facilitate the recyclability of the catalyst,^31^ or enable a continuous flow process for this particular reaction.^32,33^

The use of epoxides and carbon dioxide as starting materials further benefits from particularly high atom economies of 100% since all starting materials are fully incorporated in the product. Other synthetic routes for cyclic carbonates include transesterification or phosgenation, which suffer from issues such as a lower atom economy and the use of toxic reagents.^34,35^

Various ionic liquid-based homogeneous and heterogeneous catalysts have been reported for the conversion of common epoxides, such as propylene oxide or styrene oxide, to cyclic carbonates using different supports for ionic liquids or polymerizable ionic liquid-based precursors.^36−39^

Our studies, aiming for bio-based cyclic carbonates, are motivated by the annual production of such compounds on a multiton scale,^40^ which are used as electrolyte solutions in lithium-ion batteries^41^ or as precursors for isocyanate-free polyurethanes.^42^ This high demand highlights the necessity to develop synthetic routes utilizing bio-based feedstocks including terpenes, such as limonene, and vegetable oils, such as linseed oil, to become independent from crude oil as a limited fossil feedstock. The homogeneous catalysis of such bio-based cyclic carbonates is well-established, but only a few examples of heterogeneous catalysts exist in the literature.^43−48^ Supports based on carboxymethyl cellulose^49^ and polyethylene^50^ have been reported. However, silica is the most used support.^48,51−53^

Our previous studies,^26,28^ which investiated using silica-supported ionic liquids as heterogeneous catalysts for the formation of cyclic carbonates from epoxides, showed a synergistic effect between the ionic liquid and support material. It was also briefly mentioned that although catalytic water can be beneficial in homogeneous systems,^54^ the free hydroxyl groups and the mildly acidic surface of the silica support as well as the high affinity silica has to water can trigger the formation of undesired byproducts. After the epoxide ring is opened, byproducts such as diols, polymers, or oligomers can form.^26,28^ For this reason, there is increasing interest in finding a support material that is versatile in terms of its surface hydrophilicity and tunable in its shape and porosity as an alternative to silica.

Advanced silicon-based inorganic compounds can be obtained by the pyrolytic conversion of preceramic polymers, such as that of polysiloxanes to silicon oxycarbide (SiOC) in an inert atmosphere.^55^ The polymer-derived ceramics route^56^ yields for instance Si–O–C materials that typically consist of nanodomains rich in silicon dioxide, separated by a carbon phase.^57^ Functionalization of the preceramic polymer and the choice of the pyrolysis atmosphere and temperature offer room for property optimization to yield materials with high chemical and thermal resistance for various fields of applications.^55^ For instance, stopping the pyrolysis process at a stage where the polymer is only partly converted provides materials with intermediate characteristics of polymers and ceramics, which are called “ceramers”.^58^ Depending on the degree of conversion, ceramers can feature tunable surface hydrophilicity and a high specific surface area, rendering them suitable adsorbents,^58,59^ also for carbon dioxide.^60^

Further, polymer-derived ceramics can be processed employing advanced shaping methods including additive manufacturing^61,62^ or solidification templating.^63−67^ Solidification templating, commonly referred to as freeze-casting,^68^ is a porosification technique where a ceramic slurry or a preceramic polymer solution is cast into a mold, with a subsequent controlled solidification followed by selective removal of the solvent, e.g., via freeze-drying. This technique facilitates the generation of templated pore structures.^65^ By variation of the solid or polymer content, selection of the structure-directing solvent, and the thermal gradient applied during solidification, pore characteristics such as total porosity, pore morphology, pore orientation, and pore size distribution can be adjusted straightforwardly. The removal of the structure-directing solvent prior to thermal treatment renders solidification templating a more sustainable porosification method for obtaining monolithic catalyst supports compared to when sacrificial porogens are burned out, as the solvent can be recycled in an upscaling event.

In the case of preceramic polymer solutions, the solidification process initiates the phase separation of the solvent from the preceramic polymer. However, to retain the templated shape upon removal of the solvent and during the subsequent pyrolytic conversion process, the preceramic polymer must be cross-linked. Controlled polycondensation treatments have been employed to facilitate the freeze-casting of polysiloxanes,^64,65,69,70^ but these reactions are generally rather slow at low temperatures. Photopolymerization-assisted solidification templating, which has been shown using the thiol–ene “click” reaction,^63,66,71^ is an interesting alternative, as it is feasible at low temperatures.

With regard to the continuous production of cyclic carbonates, using macroporous monoliths as SILP supports instead of a packed bed reactor offers a high degree of control over the fluid flow,^72^ in addition to easier catalyst handling. Examples of monolithic SILPs exist in the literature,^73−76^ where ionic liquids have been immobilized on porous cellulose monoliths^73−75^ or where silane monomers have served as precursors for polymer-supported ionic liquids.^76,77^ However, to the best of our knowledge, no work thus far has reported on the physisorption of ionic liquids on silicon oxycarbide or has subsequently used such supported ionic liquids as heterogeneous catalysts for the production of cyclic carbonates.

In this paper, we present the synthesis and characterization of silicon oxycarbide-based SILPs (SiOC-SILPs) and their successful application as heterogeneous catalysts for the formation of bio-based cyclic carbonates starting from limonene oxide and epoxidized linseed oil. The catalytic performance of the SiOC-SILPs was compared to that of state-of-the-art silica-supported ionic liquids (SiO_2_-SILPs), and it was found that the developed SiOC-supported species obtained higher yields and higher selectivities. Ultimately, macroporous monolithic SiOC-SILPs were successfully applied for the selective continuous production of cyclic carbonates.

## Results and Discussion

### Preparation and Characterization of Silicon Oxycarbide as Support Material

**Figure 1:**
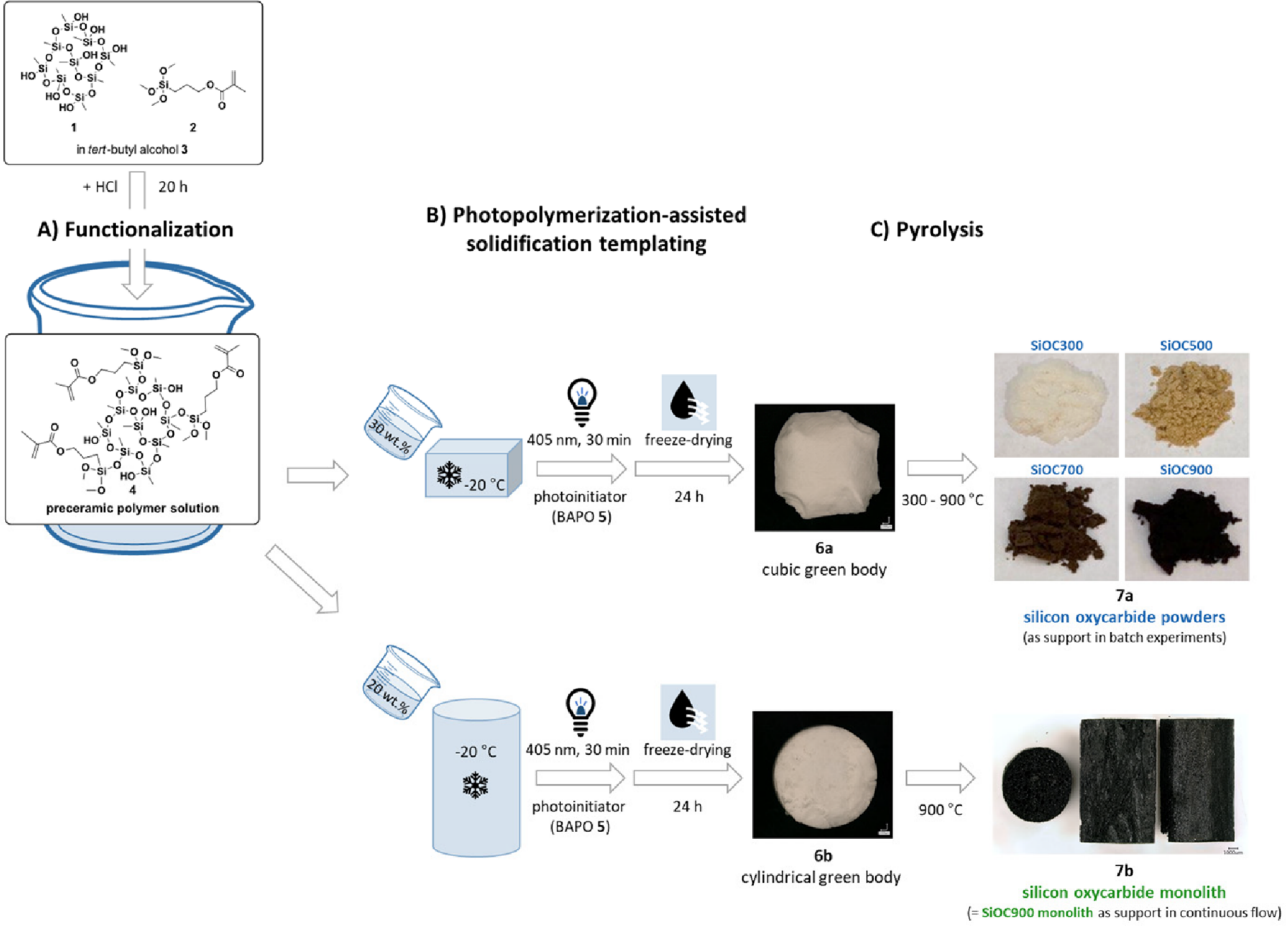
Functionalization (A) of polysilsesquioxane **1** to obtain a photocurable solution **4**, suitable for photopolymerization-assisted solidification templating (B) of polysiloxane-derived ceramics.

For the preparation of the silicon oxycarbide supports **7**, commercially available polysilsesquioxane **1** was functionalized (**A**) with photoactive methacrylate groups **2** to perform photopolymerization-assisted solidification templating (**B**), as illustrated in Figure 1. The preceramic solutions **4** with a polymer content of 20 or 30 wt % were mixed with the radical initiator phenylbis(2,4,6-trimethylbenzoyl)phosphine oxide (BAPO **5**) and frozen at −20 °C to induce phase separation of the polymer from *tert*-butyl alcohol **3**, which acted as the solvent for functionalization (**A**) and as a structure-directing agent for solidification templating (**B**). Irradiation with blue light (405 nm, 30 min) was performed at −20 °C to stabilize the templated state via a radical cross-linking reaction, yielding samples of sufficient structural integrity to further remove the frozen solvent by sublimation.

In the first step, silicon oxycarbide powders **7a** were prepared to perform batch experiments. Green bodies **6a** were converted to silicon oxycarbides **7a** via pyrolytic treatment (**C**) in an argon flow employing different pyrolysis temperatures (300–900 °C). This was followed by ball milling and sieving (90 μm mesh size) in order to obtain particle sizes comparable to silica-60. In a second step, the batchwise production of cyclic carbonates was transitioned to a continuous mode using optimized monolithic silicon oxycarbide supports **7b**.

The pyrolytic conversion was studied by thermogravimetric analysis (TGA, Figure S1), which showed a typical conversion of cross-linked polysiloxane **6** to silicon oxycarbide **7**, undergoing two distinct mass-loss steps. First, low-molecular-weight residuals from functionalization (**A**) as well as any monomers not sufficiently bound to the polymeric backbone are removed between 200 and 400 °C. An interruption of the pyrolysis in this phase still yields silicone-type compounds. The main conversion from green body **6** to silicon oxycarbide **7** occurs during the second mass-loss stage at *T* > 400 °C via dehydrogenation of the functional groups. In the range between approximately 500 and 700 °C, the evolution of volatile decomposition products usually reaches its maximum. An interruption of the conversion process in this stage yields ceramers with particularly high specific surface areas.^58^ Upon reaching 800 °C, the conversion can be considered complete. Four distinct pyrolysis temperature treatments were selected to compare the polymer state (300 °C, denoted as SiOC300) with the intermediate ceramer states (SiOC500 and SiOC700) and the amorphous ceramic state (SiOC900) for the application as a catalyst support for SILPs. The silicon oxycarbides **7a** showed a difference in color as a result of the different degrees of carbon conversion (Figure 1).

**Figure 2:**
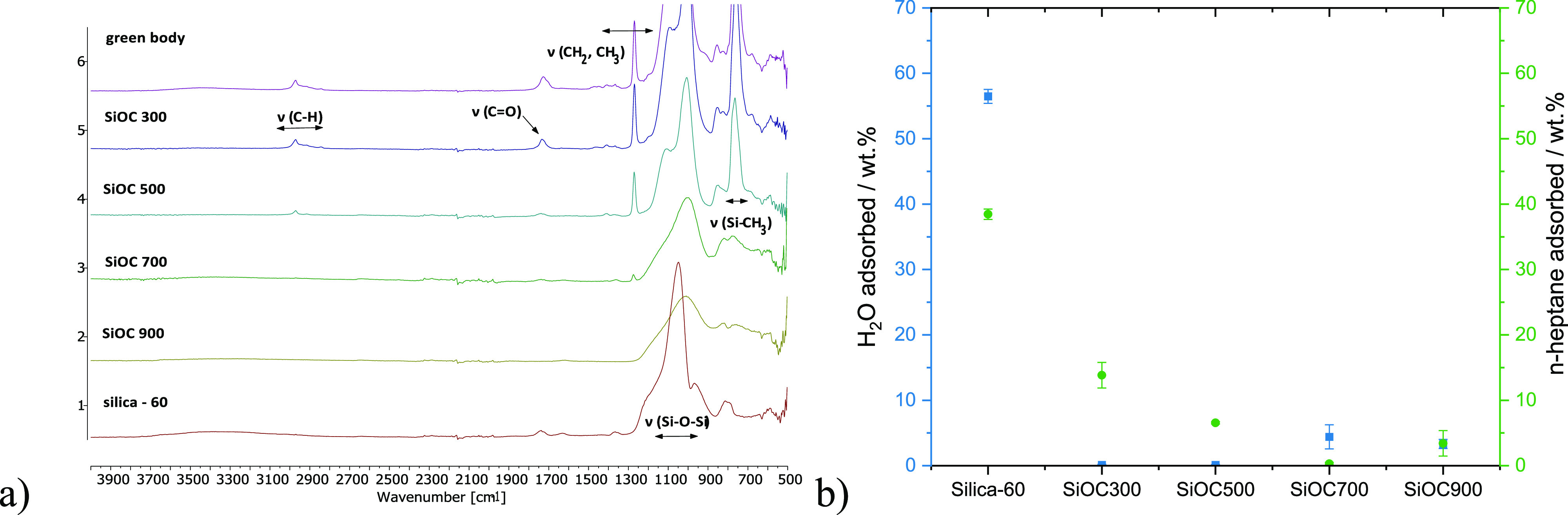
Characterization of surface functionalities and affinity to polar and nonpolar solvents of silicon oxycarbides **7a** derived from different pyrolysis temperatures compared to silica-60 via (a) FTIR spectra and (b) solvent vapor adsorption.

Fourier transform infrared spectroscopy (FTIR, Figure 2a) revealed that when pyrolysis was stopped at 300 °C, methacrylate moieties were still present, while at 500 °C, they had been converted (C=O band at 1728 cm^–1^). At 700 °C, alkyl groups had been almost completely converted as well (CH: 3027–2811 cm^–1^; CH_2_, CH_3_: 1505–1228 cm^–1^). Moreover, the Si–CH_3_ groups were converted successively at higher temperatures (759 cm^–1^). At 900 °C, the preceramic polymer was fully converted to an amorphous silicon oxycarbide ceramic, preserving the Si–O–Si bands at 1214–901 cm^–1^. Ionic liquid loading onto the silicon oxycarbide supports **7a** led to additional vibrations at 3023–2762 cm^–1^ and 1502–1340 cm^–1^, indicating C–H and C–C vibrations of the tetrabutylammonium cation (Figures S7 and S8).

Volatiles from the decomposition of methacrylate and alkyl groups, such as hydrocarbons and hydrogen,^55,58^ led to high specific surface areas (Figure S3 and Table S1) in the intermediate processing stages, with a maximum of 550 m^2^ g^–1^ in microporous SiOC500. In SiOC900, transient microporosity disappeared, indicated by a low specific surface area of 2 m^2^ g^–1^. In this case, impregnation with ionic liquid only slightly increased the specific surface area to 2–4 m^2^ g^–1^. At an ionic liquid loading of 20 wt %, the microporosity in the SiOC500 and SiOC700 ceramers can be assumed to be filled. However, both of the best-performing catalysts, **SILP 5a** and **SILP 11a**, are nonmicro/nonmesoporous materials according to nitrogen adsorption measurements. In contrast, upon loading mesoporous silica-60 with ionic liquid, the surface area decreased but remained at a high level of 221–263 m^2^ g^–1^.

Furthermore, the affinity of the SiOC support materials to polar and nonpolar molecules was investigated by solvent adsorption and compared to silica-60, a commonly used support material for SILPs. Water and *n*-heptane are substances with mainly polar and nonpolar interactions, respectively, and can be used to quantify the hydrophilicity of a material.^60^ The amount of solvent adsorbed was measured gravimetrically after exposure of the dried materials to saturated atmospheres at 25 °C for 24 h. Silica took up an exceptional amount of water (57 ± 1 wt %) and *n*-heptane (39 ± 1 wt %) (Figure 2b). The higher uptake of water compared to that of *n*-heptane is indicative of the material’s hydrophilicity, which is related to the oxidic surface. In contrast, the silicon oxycarbides took up significantly lower amounts of solvent vapor. Further, a trend of decreasing affinity to *n*-heptane and increasing affinity to water was observed with increasing pyrolysis temperatures. This transition from hydrophobic to hydrophilic characteristics resulted from a successive loss of residual polymer functionalities, as confirmed by FTIR (Figure 2a). The observed transition temperature for the transition from being hydrophobic to slightly hydrophilic was between 500 and 700 °C, which is in accordance with the results from a previous study.^78^

In comparison to silica-60, the incorporated carbon in the silicon oxycarbide pyrolyzed at 900 °C (14.9 ± 0.5 wt % in SiOC900, determined by the combustion method) and the lack of mesoporosity lowered the affinity of this material to water vapor, which is beneficial for the selective formation of cyclic carbonates, as shown in Figure 4b and discussed in detail hereinafter.

Based on literature procedures,^26^ the powdered silicon oxycarbides **7a** pyrolyzed at different temperatures (SiOC300, SiOC500, SiOC700, SiOC900) and silica-60 (SiO_2_) were impregnated with tetrabutylammonium chloride (TBAC **8**), tetrabutylammonium bromide (TBAB **9**), and tetrabutylammonium iodide (TBAI **10**) to generate SILPs that were then used further as heterogeneous catalysts (Table 1).

**Table 1:**
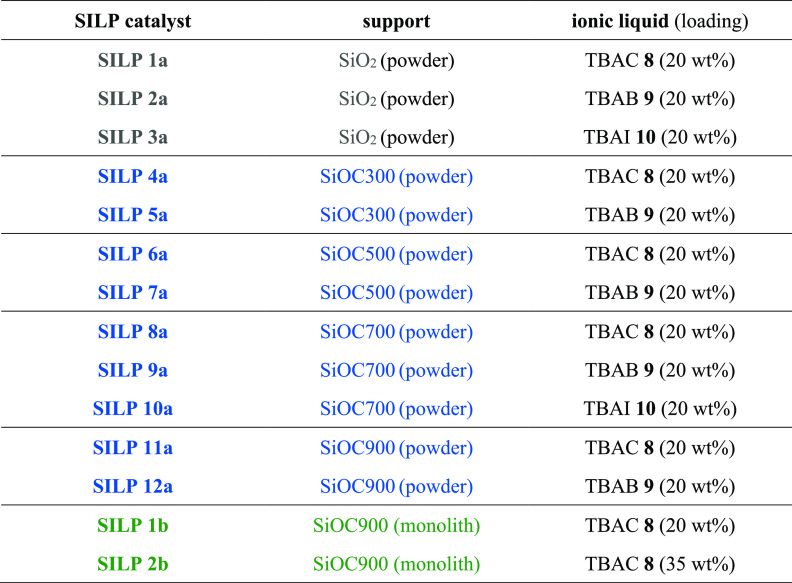
Supported Ionic Liquids: Powdered and Monolithic Supports, Impregnated with Ionic Liquids, Were Employed for the Formation of Bio-Based Cyclic Carbonates in Batch Mode and Continuous Flow

X-ray photoelectron spectroscopy (XPS) spectra were recorded to determine the types of chemical bonding and functional groups present on the surfaces of the SiOC-based SILPs compared to those present on the SiOC support materials. The survey scans revealed the existence of Si, C, and O on the SiOC surfaces and additional Cl and N for the SILPs (Figure S9 and Table S2). Peak assignments of the high-resolution core-level spectra were based on the literature.^79^ The C 1s fits (Figure S11) showed C–C bonds (sp^3^ hybridized) decreasing in intensity upon increasing pyrolysis temperature and increasing in intensity by the addition of ionic liquid. Deconvolution of the Si 2p spectra (Figure S13) revealed various binding states, whereas peak assignment was challenging due to overlapping spin–orbit components. The N 1s and Cl 2p core-level spectra were present only in the SILPs (Figures S12 and S14). The O 1s spectra revealed the presence of C–O, Si–O, and Si–O–Si bonds (Figure S10). Furthermore, differences in the structural behavior of the SILPs and supports could be inferred, especially from the O 1s spectra. This suggested an interaction of the catalytically active ionic liquid, especially with the oxygen atoms of the SiOC support.

To investigate the stability of the SILPs, thermogravimetric analyses of the ionic liquids, SiO_2_-SILPs, and SiOC-SILPs were conducted in air (Figure S6). Measurements revealed that after an initial loss of water (25–100 °C), mass loss started at around 160 °C, independent of the supporting material or kind of ionic liquid, showing the high thermal stability of the ionic liquids that were used. In addition, complete degradation of the ionic liquid was observed at around 230 °C for the silicon oxycarbide-based SILPs, while for the silica-based SILPs, the end of degradation was observed at 270 °C, most likely due to a stronger interaction of the support surface with the ionic liquid or the degradation products. Furthermore, the same thermal stabilities with regard to the onset of decomposition for the supported and unsupported ionic liquids were observed. As expected, further conversion processes were observed for SiOCs previously pyrolyzed at 300 °C (SiOC300).

### Comparison of Silicon Oxycarbide- and Silica-Based Catalysts for the Formation of Bioderived Cyclic Carbonates **15** and **18**

**Figure 3:**
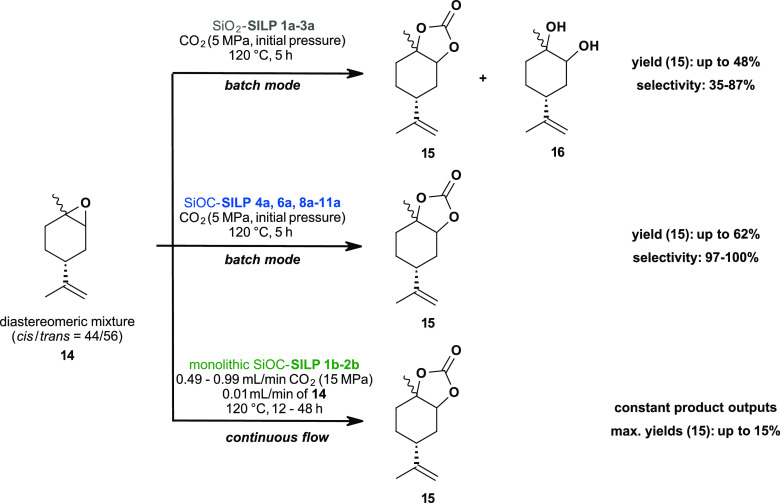
Limonene carbonates **15**: SiO_2_-SILPs and SiOC-SILPs were used as catalysts in batch mode and in continuous flow.

Limonene oxide **14** was chosen as the primary bio-based feedstock since its potential as a feedstock for bulk chemicals, such as cyclic carbonates, has been displayed in a global annual production of 43 Mt and a global market of $314 million USD (2020).^80^ SiO_2_-SILPs for the batch and continuous production of limonene carbonate (Figure 3) have already been studied by our group.^26^ Furthermore, the production of linseed oil carbonates **18** as promising precursors for nonisocyanate polyurethanes^51^ is presented (Figure 5).

Based on reports by Morikawa et al.,^81,82^ in which tetrabutylammonium halides **8**–**10** were screened in batch mode for the conversion of limonene oxide **14** to limonene carbonate **15**, we expanded the scope of preliminarily tested homogeneous catalysts to imidazolium-based ionic liquids (Table S3) in a previous work.^26^ During this screening, tetrabutylammonium halides **8**–**10** turned out to be suitable catalysts for the formation of limonene carbonate **15**, resulting in a selective conversion of 72% and a yield of 68% (isolated yield of 57%) after 20 h of the reaction at 100 °C in the case of the best candidate, TBAC **8** (Table S3, entries S1–S3). Subsequently, TBAC **8** was physisorbed on silica as the most commonly used support material for SILP catalysts and employed for the continuous production of different limonene carbonates. During the optimization of the continuous formation process in the previous work, 120 °C turned out to be the optimum temperature in terms of yields, while higher temperatures were found to lead to the degradation of the catalyst. Due to this higher reaction temperature, the reaction time could be shortened from 20 to 5 h for the catalyst screening performed in this work, resulting in a conversion of 58% of limonene oxide **14** using TBAC **8** (Table S4, entry S7). Furthermore, we were interested in the investigation of both isomers starting from the commercially available *cis*/*trans* mixture; however, the more reactive *trans* isomer (*cis*: 23%, *trans*: 85%) can be obtained via kinetic separation^83^ to further increase yields.

**Figure 4:**
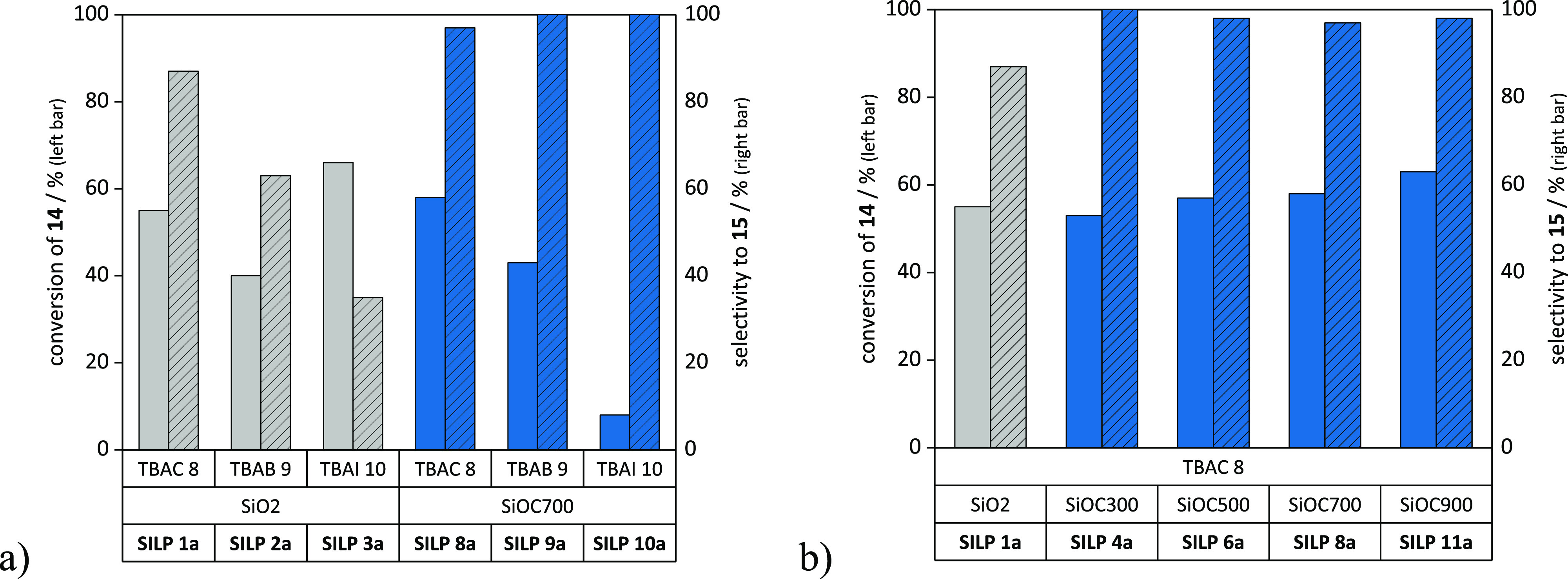
Catalyst screening for the formation of limonene carbonate **15** in batch mode. Conditions: 0.5 mmol of **14** (*cis*/*trans* = 43/57), SILP catalyst (0.05 mmol of **8**–**10**, catalyst loading: 20 wt %), 120 °C, 5 MPa of CO_2_ (gaseous, initial pressure), 5 h. Further details about the determination of the conversion and selectivity (ratio of yield and conversion) are given in the Supporting Information, section S.3.1.

Commonly used SiO_2_-based SILPs impregnated with tetrabutylammonium halides **8**–**10** were employed as heterogeneous catalysts and compared to the SiOC-based SILPs. Out of all of the tested SiO_2_-based **SILPs 1a**–**3a** (Figure 4a), **SILP 1a**, impregnated with TBAC **8**, performed the best, following the nucleophilicity of halides in an aprotic polar environment (Cl^–^ > Br^–^ > I^−^). Nevertheless, the yield dropped from 55% to 48%, and the selectivity decreased from 95% to 87% when compared to the homogeneous version (Table S4, entry S7). Moreover, it was observed that the selectivity decreased further to 63% and 35% when bromide and iodide served as anions in the SiO_2_-based **SILPs 2a** and **3a**, respectively (Figure 4a).

Residual water and free hydroxyl groups on the surface of silica triggered the ring opening of the epoxide and the formation of diol **16** in amounts of 3–17%, determined via gas chromatography–mass spectrometry (GC/MS). Furthermore, when 10 mol % of water was added to the reaction, generating a polar protic environment, decreases in conversion from 55% to 50% and selectivity from 87% to 66% were observed (Table S4, entry S10).

The co-catalytic effect of silica on the formation of undesired byproducts was further verified when silica was solely employed as a catalyst (Table S4, entry S22). In this case, conversion reached 36%, and 13% of diol **16** and no carbonate **15** were formed. This experiment showed the inevitable influence of the silica surface and the requirement for the development of alternative supports for the ionic-liquid-catalyzed heterogeneous conversion of epoxides to cyclic carbonates to increase selectivity.

The diol **16** was no longer formed when powdered silicon oxycarbides **7a** were employed as support materials, displayed by the obtained selectivities of 97–100% (Figure 4b) and increased yields of 53–62%. This correlated with the lower affinity of silicon oxycarbides for water, as was made apparent from the solvent vapor adsorption experiments (Figure 2b). Moreover, the addition of 10 mol % of water to the reaction mixture resulted in no change in the conversion and selectivity (Table S4, entry S21) when SiOC was employed as the support. In comparison, both the conversion and the selectivity decreased when employing silica-based SILPs with catalytic amounts of water (Table S4, entry S10), as mentioned previously. Therefore, the constantly high conversion and selectivity obtained with the SiOC support suggests the involvement of the mildly acidic surface of silica on the epoxide ring opening and the subsequent formation of diol **13** as byproduct.

The trend of nucleophilicity (Cl^–^ > Br^–^ > I^–^) was preserved with SiOC-**SILPs 8a**–**10a**, displayed by the obtained yields of 8–56%. Additionally, the influence of the pyrolysis temperature (300–900 °C) of the silicon oxycarbide supports was investigated (Figure 4b). Yields increased from 53% to 62% with higher pyrolysis temperatures, and remarkable selectivities of 97–100% were obtained without leaching of the catalyst (limit of detection: 0.1 mg, ≤0.2% of total amount of TBAC **8**). Furthermore, the silicon oxycarbide supports did not exert any catalytic effect on their own (Table S4, entries S23–S26), in contrast to the silica support (Table S4, entry S22).

Overall, **SILP 11a** (20 wt % TBAC **8**, SiOC900) exhibited the highest yield and a selective formation of limonene carbonate **15** with 100% atom economy; thus, this material was subsequently employed in continuous flow experiments in monolithic form **7b**.

**Figure 5:**
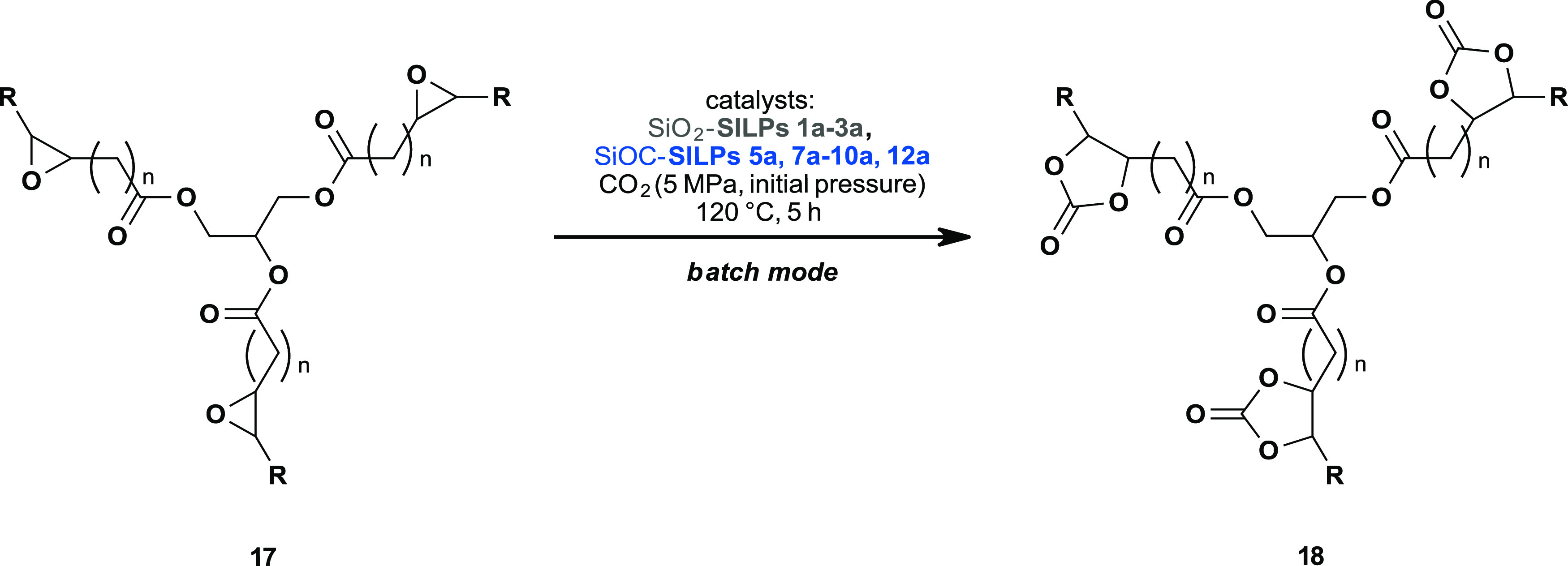
Linseed oil carbonates **18**: SiO_2_-SILPs and SiOC-SILPs as catalysts in the batch mode.

Furthermore, we investigated the reaction of epoxidized linseed oil **17** catalyzed by SiO_2_-SILPs and SiOC-SILPs (Figure 5). Conversion was determined via proton nuclear magnetic resonance (^1^H-NMR; see the Supporting Information, section S.3.2) by quantifying the signal of the epoxide moieties (δ = 3.21–2.82 ppm) and using the protons next to the carbonyl groups of the backbone (δ = 2.29 ppm) as the internal standard. Yields could not be determined due to the overlapping signals in the ^1^H-NMR spectra (carbonate: δ = 5.40–4.06 ppm). However, the formation of carbonate **18** was further proven via FTIR (Figure S23), where the appearing C=O band at 1792 cm^–1^ indicated the ongoing reaction.^51^

An extensive homogeneous catalyst screening with various ionic liquids was reported by Tassaing et al.,^84^ where the influence of halides (Cl^–^, Br^–^, I^–^) and cationic cores (e.g., ammonium, phosphonium, sulfonium, imidazolium, and pyridinium) on the conversion of epoxidized linseed oil **17** was investigated. It was revealed that different halides have the most pronounced impact on the catalytic activity. Tetrabutylammonium halides **8**–**10** turned out to be promising candidates, resulting in yields of 17–30% (TBAB **9** > TBAI **10** > TBAC **8**) on a 30 g scale (100 °C, 10 MPa).

Reproduction on a 220 mg scale (120 °C, 5 MPa) resulted in the same order of catalytic activity (TBAB **9** > TBAI **10** > TBAC **8**), displayed in yields of 41–56% (Table S6, entries S32–S34). Almost complete conversion (97%) was achieved after 20 h. The order of catalytic activity clearly shows the competition between the nucleophilicity and the size of the anion since epoxidized linseed oil **17** is a sterically demanding substrate and smaller anions can reach the epoxide moieties easier.

**Figure 6:**
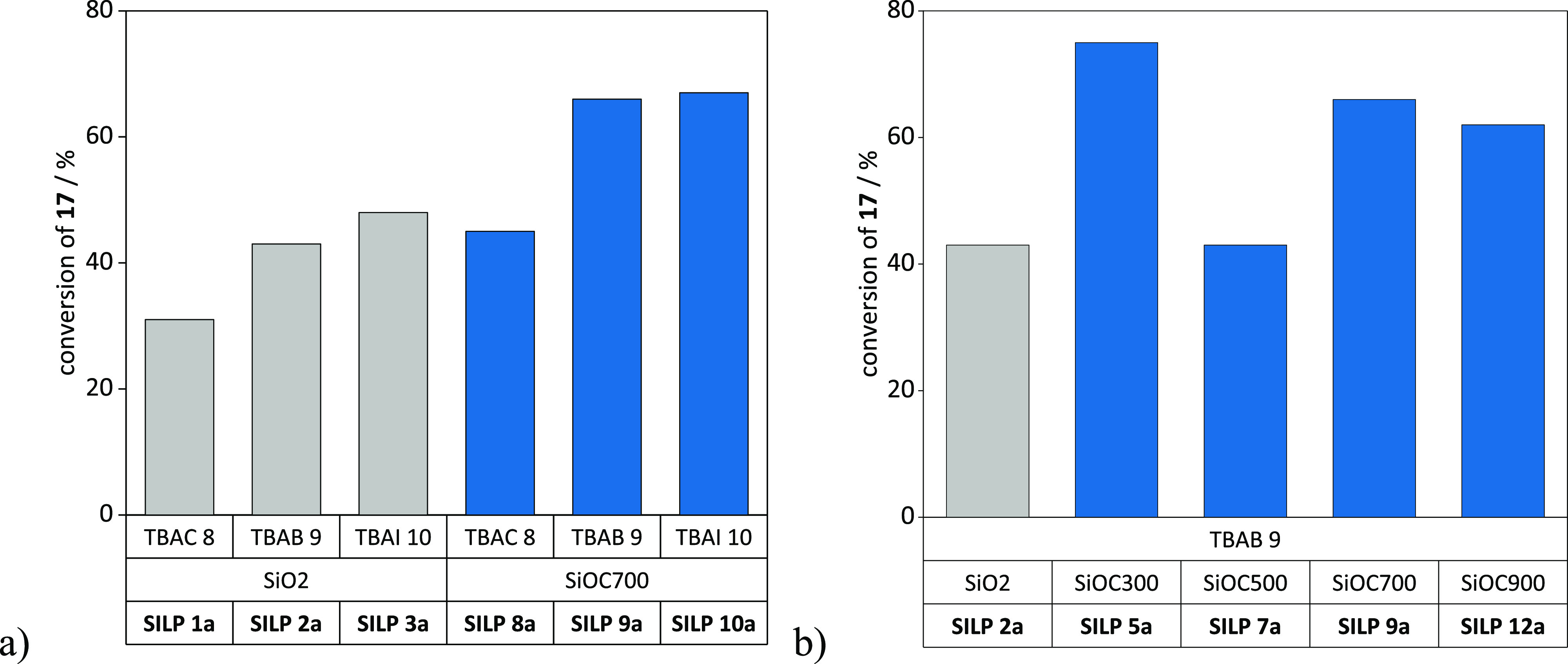
Catalyst screening for the formation of linseed oil carbonates **18** in batch mode. Conditions: 220 mg of **17**, SILP catalyst (0.02 mmol of **8**–**10**, catalyst loading: 20 wt %), 120 °C, 5 MPa of CO_2_ (gaseous, initial pressure), 5 h. Further details about the determination of conversion are given in Supporting Information, section S.3.2.

For SiO_2_-**SILPs 1a**–**3a** (Figure 6a), impregnation with TBAI **10** resulted in the highest conversion of 48%. Conversion increased to 66–67% with SiOC-**SILPs 9a** and **10a** (Figure 6a) impregnated with TBAB **9** and TBAI **10**. Yields were further increased by employing the SiOC-SILPs prepared with the silicon oxycarbide supports derived from different pyrolysis temperatures (300–900 °C), achieving conversions of up to 75% with **SILP 5a** using support SiOC300 (Figure 6b). Finally, it was proven that all supports showed only low or no co-catalytic effect (Table S6, entries S46–S50). The leaching of catalysts **8**–**10** in batch experiments could not be determined due to the inseparability of the SILP catalyst from the highly viscous and poorly soluble reaction mixture, e.g., via centrifugation or via dissolution of the reaction mixture in apolar solvents.

The interaction behavior of the SiOC supports^79^ with ionic liquid was further investigated via XPS, as discussed previously. A comparison of the O 1s spectra of the supports and the SILPs (Figure S10) revealed different interaction behavior and, thus, an interaction of the catalyst and support predominantly via the oxygen atom. This suggests that the interaction of the oxygen of the SiOC support with the ionic liquid is of crucial importance for the formation of cyclic carbonates using SiOC-supported ionic liquids compared to homogeneous catalysis.

Ultimately, studies in continuous flow with the monolithic silicon oxycarbide-supported ionic liquids were conducted solely with limonene oxide **14**, as neat epoxidized linseed oil **17** turned out to be too viscous for continuous transport; the addition of necessarily polar solvents for dissolution of epoxidized linseed oil **17** led to significant catalyst leaching (30%).

### Characterization of Monolithic SiOC-**SILPs 1b** and **2b**

**Figure 7:**
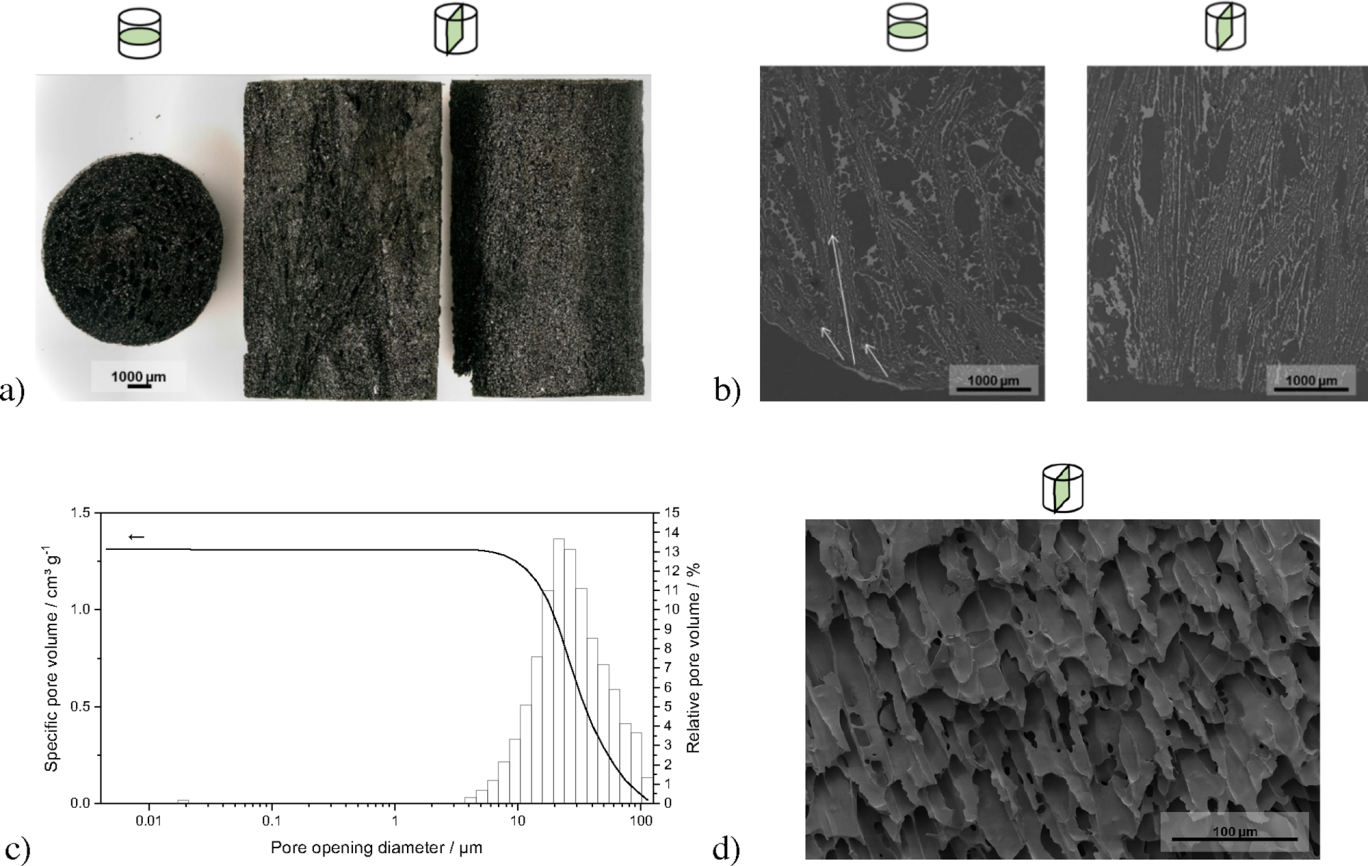
Characterization of monoliths **7b** for continuous experiments: (a) optical microscopy images; (b) scanning electron micrographs (backscattered electron detection) of sections embedded in epoxy resin, ground, and polished; (c) mercury intrusion porosimetry pore size distribution; and (d) scanning electron micrograph of a fracture surface (field emission gun–scanning electron microscopy, secondary electron detection).

In Figure 7a, an optical microscopy image of monolithic silicon oxycarbide pyrolyzed at 900 °C **7b** cut in cross and longitudinal sections with regard to the flow direction in the reactor for continuous experiments is shown. The pores are further visualized by the color contrast of silicon oxycarbide and epoxy resin, which appear as bright gray and dark gray, respectively, due to their difference in atomic mass, with the aid of backscattered electron detection in scanning electron microscopy (Figure 7b; method details are given in SI section S.7.1). Evidently, the porosity generated by solidification templating in aluminum molds resulted in radial pore orientation in the cross section due to the preferential nucleation of *tert-*butyl alcohol **3** on aluminum that features high thermal conductivity, thereby causing oriented crystal growth from the outside to the inside of the cylinders, as indicated by the arrows in Figure 7b. Further characterization of the monolith by mercury intrusion porosimetry revealed a broad macropore size distribution with a median pore opening diameter of 26 μm (Figure 7c). Observing a fracture surface of the monolith revealed the prismatic pore morphology templated by solidified *tert-*butyl alcohol **3** (Figure 7d).

The permeability of silicon oxycarbide monoliths **7b** was quantified by applying Forchheimer’s equation for compressible fluids (Formula S1 and SI section S.2.2)^72^ to obtain both *k*_1_ (Darcian) and *k*_2_ (non-Darcian) permeability constants from measured pressure drops and permeating air flow using filtered compressed air as the permeating fluid (Figure S4). The air flow was applied in the same flow direction as that in continuous carbonate production. The Darcian flow refers to a linear dependence of the fluid flow on the pressure gradient and is solely dependent on the fluid’s dynamic viscosity. Non-Darcian permeability also considers inertial resistance, which becomes particularly relevant for applications in which fluids of high density are used. The determined permeability constants of monoliths **7b** (*k*_1_ = 10^–11^ m^2^, *k*_2_ = 10^–6^ m) are classified in the order of honeycomb or fibrous filters.^72^

**Figure 8:**
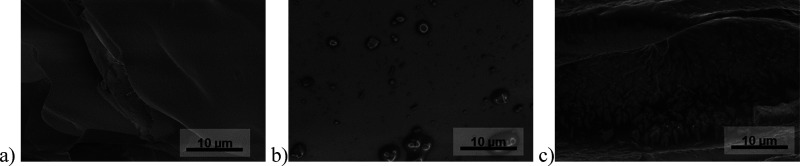
Scanning electron micrographs of fracture surfaces (field emission gun–scanning electron microscopy, secondary electron detection): (a) monolithic silicon oxycarbide **7b**, (b) **SILP 1b** (20 wt % TBAC **8**), and (c) **SILP 2b** (35 wt % TBAC **8**). Images of an additional spot and additional magnifications are given in Figure S5.

For the physisorption of amounts of 20–35 wt % ionic liquid TBAC **8**, silicon oxycarbide monoliths **7b** were added to a methanolic solution of TBAC **8** and treated in an ultrasonic bath for efficient removal of air from the macropores. After solvent removal, surface morphologies of the fractured monoliths were investigated by high-resolution field emission gun–scanning electron microscopy (FEG-SEM, method details are given in SI section S.7.1), which revealed that the physisorbed ionic liquid caused a change in the surface morphology, as shown in Figure 8. In comparison to the uncoated reference material (monolithic silicon oxycarbide **7b**), partial coverage of the monolith’s inner surface with ionic liquid was observed for lower loadings (20 wt % TBAC **8**, **SILP 1b**), while a more uniform distribution was achieved for higher loadings (35 wt % TBAC **8**, maximal loading, **SILP 2b**) (Figure 8).

For continuous application, **SILPs 1b** and **2b** were finally inserted in a shrinking tube, which was heated carefully with a heat gun to shrink tightly onto the monoliths to perfectly fit stacked monoliths into a catalyst cartridge and thus ensure flow through the macropores of the monolithic SiOC-SILPs.

### Continuous Formation of Limonene Carbonate **15** with Monolithic SiOC-**SILPs 1b** and **2b**

**Figure 9:**
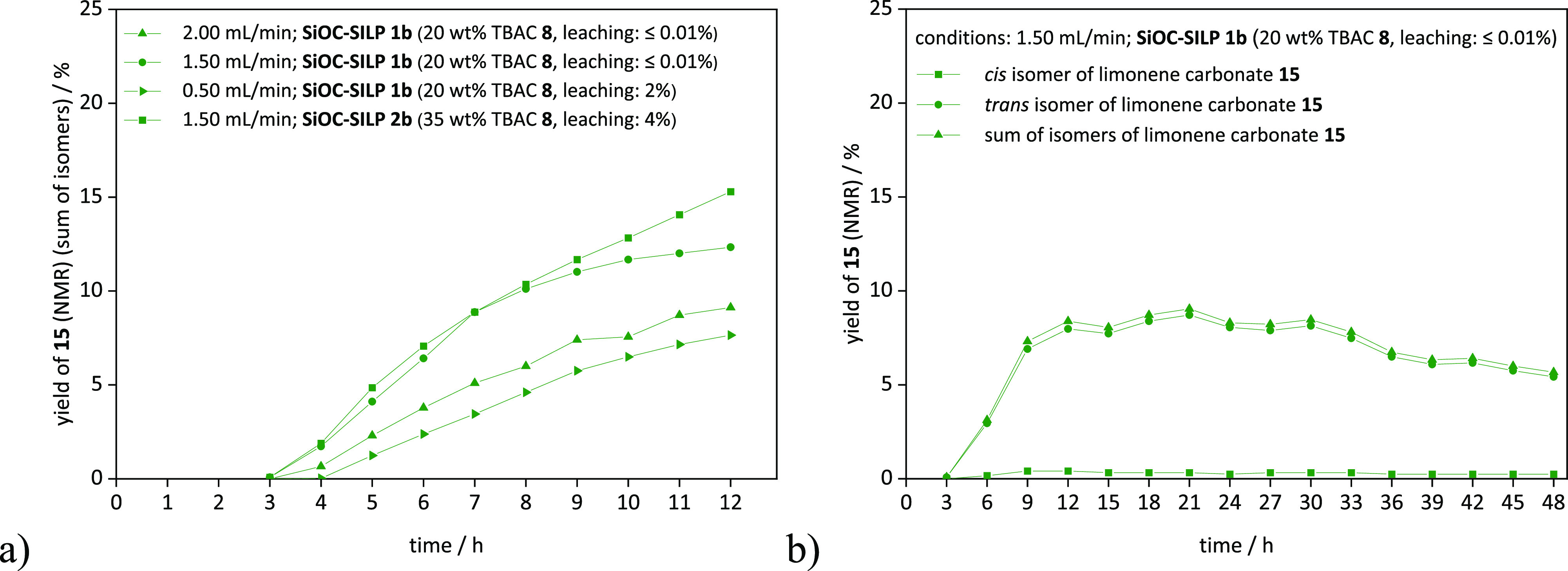
Continuous production of limonene carbonate **15** with monolithic SiOC-**SILPs 1b** and **2b**. Conditions: 0.01 mL/min of limonene oxide **14** (*cis*/*trans* = 43/57), **SILP 1b** and **2b** (15–20 mm monolith pieces, 220 mm in total), 1.99–0.49 mL/min of CO_2_ (15 MPa), at 120 °C for (a) 12 h or (b) 48 h. Maximum and overall yields are depicted in Table S5.

With the monolithic SiOC-**SILPs 1b** and **2b** in hand, the formation of limonene carbonate **15** in continuous flow was conducted (Figure 9) by using a device normally employed for supercritical carbon dioxide applications^26^ (details are given in the Experimental Section and in SI section S.7.1). In the course of the continuous flow experiments, the influences of the flow rate of carbon dioxide and the catalyst loading were investigated. As illustrated in Figure 9, all experiments resulted in a desired constant product output, independent of the varied parameters.

An initial experiment employing a flow rate of carbon dioxide of 1.49 mL/min (total flow: 1.50 mL/min) and **SILP 1b** (Figure 9a) as the catalyst resulted in a maximum yield of 12%. Only traces of leached catalyst (limit of detection: 0.1 mg, ≤0.1% of total amount of TBAC **8**) were observed. A higher flow rate of carbon dioxide of 1.99 mL/min (total flow: 2.00 mL/min) led to a decrease in the maximum yield to 9% (Table S5, entry S27) due to a shorter residence time of 7.6 min as opposed to 10.1 min at a total flow of 1.50 mL/min.

However, a lower flow rate of carbon dioxide of 0.49 mL/min (Table S5, entry S29; total flow: 0.50 mL/min; residence time: 30.5 min) resulted in a decrease of the maximum yield to 8% and a catalyst leaching of 2%. This indicates that a minimum concentration of carbon dioxide is required for sufficient carbonate formation and that a higher flow rate is beneficial for removing reacted species from the catalytically active surface.

Moreover, a higher catalyst loading of 35 wt % (**SILP 2b**) resulted in a higher maximum yield of 15% (Table S5, entry S30), whereupon the full maximum was not reached even after 12 h of reaction time (Figure 9a). However, 4% catalyst leaching was observed; thus, a decrease of the yield over time can be expected. Ultimately, a stability test over 48 h using **SILP 1b** (total flow: 1.50 mL/min) resulted in an overall yield of 7% without leaching of the catalyst (limit of detection: 0.1 mg, ≤0.1% of total amount of TBAC **8**) (Figure 9b).

## Conclusions

In this paper, we investigated SiOC-SILPs for their application as heterogeneous catalysts in the formation of bio-based cyclic carbonates **15** and **18** and compared them to the performance of commonly used silica-based SILPs. The processing benefits associated with the polymer-derived ceramic route enabled a smooth transition from batch to continuous flow operation utilizing monolithic SiOC-SILPs.

Extensive catalyst screening in batch mode revealed that excellent selectivities of 97–100% and yields of 53–62% after 5 h of reaction time with SiOC-**SILP 4a** and SiOC-**SILPs 7a**–**9a** could be achieved for limonene carbonate **15**. In contrast, SiO_2_-SILPs resulted in a low selectivity of 87% and a yield of 48% due to the formation of diol **16** as a byproduct, which was triggered by residual water and free hydroxyl groups on the surface of silica-60. For linseed oil carbonates, yields could be increased from 48% (SiO_2_-**SILP 3a**) to 75% (SiOC-**SILP 5a**).

Finally, silicon oxycarbide monoliths were successfully implemented for continuous and selective limonene carbonate formation, resulting in constant product outputs. Studying the long-term behavior of the monolithic catalyst over 48 h resulted in an overall yield of 7% and high selectivity without significant leaching of the ionic liquid from the monolithic silicon oxycarbide.

These results illustrate that scaleable SiOC-SILP-catalyzed continuous flow processes are highly suitable for the formation of various cyclic carbonates. An extended ionic liquid screening could further lead to an increase in reaction yields. Moreover, the pore orientation within the silicon oxycarbide monoliths could be improved via rotational freeze-casting^85^ or by unidirectional freeze-casting.^86^ In conclusion, solidification-templated polymer-derived ceramics offer plenty of possibilities to tailor the porosity and surface properties to host ionic liquids, rendering silicon oxycarbide-supported ionic liquids highly interesting for future applications in heterogeneous catalysis.

## Experimental Section

Further details regarding the preparation and characterization of support materials and SILPs as well as the materials, methods, and typical procedures can be found in the SI.

### Preparation of Silicon Oxycarbide Supports via Photopolymerization-Assisted Solidification Templating

Preparation of the photocurable polysiloxane solution **4** was performed according to a modification of the procedures reported in the literature^61,62,87^ for additive manufacturing. For a typical master batch of 40 wt % polymer content, 15 g of methyl silsesquioxane **1** was dissolved in 30 g of *tert*-butyl alcohol **3** at 40 °C. Then, 5 g of 3-(trimethoxysilyl)propyl methacrylate **2** was added, and the mixture was stirred for 1 h at room temperature. One drop of concentrated HCl (37%) was added while stirring the mixture at 500 rpm. The mixture was stirred at 200 rpm for 20 h to ensure complete functionalization.

The master batch of 40 wt % polymer content was diluted with *tert*-butyl alcohol **3** to yield 10, 20, or 30 wt % polymer content in the preceramic solution **4**. Subsequently, phenylbis(2,4,6-trimethylbenzoyl)phosphine oxide **5** was added as a photoinitiator (1 wt % with respect to the polymer content). The solution was homogenized (4 min, 2000 rpm) and degassed (10 min, 800 rpm) using a planetary mixer (Thinky ARE-250).

The solution was frozen at −20 °C for 24 h in polyethylene or aluminum molds to yield cubic or cylindrical samples. The solidified samples were demolded and illuminated with a wavelength of 405 nm (Sovol, 6 W) for a total of 30 min at −20 °C. Subsequently, the samples were stored at −20 °C overnight. Solidified *tert*-butyl alcohol **3** was removed using a freeze-dryer (CHRIST Alpha 1-4 LDplus) for 21 h at 1 mbar, followed by 3 h at <0.4 mbar. Pyrolytic conversion was conducted in a tube furnace (STF 166, Carbolite) under an argon flow (30 L/h). The maximum pyrolysis temperature ranged from 300 to 900 °C (heating rate: 1 K/min; dwell time: 1 h; cooling rate to room temperature: 2 K/min). Linear shrinkage and ceramic yield were evaluated by measuring the diameter, height, and weight of the monoliths before and after pyrolysis. For the batch conversion experiments, the porous supports were ball-milled and sieved (mesh size of 90 μm). For the continuous conversion experiments, cylindrically shaped monoliths were used and impregnated with ionic liquid, as further described in the SI, section S.7.3.

### Formation of Limonene Carbonate **15** in Batch Mode

The synthesis was performed according to a modification of a literature procedure.^26^ An 8 mL glass vial was charged with limonene oxide **14** (*cis*/*trans* = 43/57, 103 μL, ρ = 0.93 g/mL; remaining amount after sampling for ^1^H NMR: 82 μL, 0.5 mmol) and internal standard naphthalene (1.3 mg, 0.01 mmol). Then, a 21 μL sample was taken to record a ^1^H NMR spectrum at *t* = 0 (Figure S15). After sampling, SiOC-**SILP 11a** (for preparation details, see SI section S.7.2; 70 mg, 0.05 mmol of TBAC **8**) was added. The glass vial, equipped with a screw cap with a septum and a cannula, was placed in a 40 mL autoclave. After pressurizing with carbon dioxide (5 MPa), the reaction mixture was stirred for 5 h at 120 °C. After completion of the reaction, the autoclave was cooled to room temperature, the carbon dioxide was released, and the reaction mixture was homogenized with 0.5 mL of deuterated chloroform. A ^1^H NMR spectrum was recorded (*t* = 5 h, Figure S15) revealing a selective conversion of 63% (62% yield).

### Formation of Linseed Oil Carbonate **18** in Batch Mode

The synthesis was performed according to a modification of a literature procedure^84^ and similarly to the formation of limonene carbonate **15**. In this case, 220 mg of epoxidized linseed oil **17** and SiOC-**SILP 5a** (27 mg, 0.02 mmol of TBAB **9**) were used, resulting in 75% conversion. Determination of conversion was performed via ^1^H NMR (see SI section S.3.2).

### Continuous Production of Limonene Carbonate **15**

Monolithic SiOC-**SILP 1b** and **2b** (for preparation details, see SI section S.7.3; 220 mm, 15–20 mm pieces) were loaded into a shrinking tube. A heat gun was used for shrinking. The sheathed monoliths were inserted into the catalyst cartridge, which was connected to a device used for supercritical carbon dioxide applications (for details, see SI section S.7.1 and ref (26)). The flow rate was set to 0.01 mL/min for limonene oxide **14** and 0.49–1.99 mL/min for carbon dioxide (15 MPa back pressure), and the catalyst cartridge was heated to 120 °C. Limonene carbonate **5a** dissolved in the starting material was collected in vials in different fractions for 12 h (one fraction/hour).

For the determination of the NMR yields, 10.0 ± 0.1 mg of naphthalene was added to each fraction. After homogenization with 0.5 mL of deuterated chloroform, an aliquot was taken for ^1^H-NMR measurements. The spectrum was compared to a reference spectrum of a mixture of 558 mg of limonene oxide **14** (0.6 mL/h, ρ = 0.93 g/mL) and 10 mg of naphthalene (see also SI section S.3.1).

## Supporting Information Available

Preparation and analysis of supports and SILPs; determination of NMR yields and conversions; catalyst screening for SILPs; materials, methods, and typical procedures; and analysis of the cyclic carbonates (PDF)

## Abbreviations

BET: Brunauer–Emmett–Teller
FEG-SEM: field emission gun–scanning electron microscopy
NMR: nuclear magnetic resonance
PSO: polysiloxane
SiO_2_: silica
SILP: supported ionic liquid phase
SiOC: silicon oxycarbide
TBAX: tetrabutylammonium halide
TGA: thermogravimetric analysis
XPS: X-ray photoelectron spectroscopy
