# Supplementary material for: Silicon Oxycarbide (SiOC)-Supported Ionic Liquids: Heterogeneous Catalysts for Cyclic Carbonate Formation
Source: ACS Sustain Chem Eng. 2024 Jan 18;12(4):1455–67. doi: 10.1021/acssuschemeng.3c05569 (PMC10829049; doi:10.1021/acssuschemeng.3c05569)
Supplement: Supplementary file 1 — sc3c05569_si_001.pdf [file sc3c05569_si_001.pdf]

Supporting Information

Silicon Oxycarbide (SiOC) Supported Ionic Liquids: Heterogeneous Catalysts for Cyclic Carbonate Formation

Philipp Mikšovsky [1, †], Katharina Rauchenwald [2, †], Shaghayegh Naghdi [3], Hannah Rabl [3], Dominik Eder [3], Thomas Konegger [2,] and Katharina Bica-Schröder [1,*]*

* katharina.schroeder@tuwien.ac.at

* thomas.konegger@tuwien.ac.at

[1]Institute of Applied Synthetic Chemistry, TU Wien, Getreidemarkt 9, 1060 Vienna, Austria.

[2]Institute of Chemical Technologies and Analytics, TU Wien, Getreidemarkt 9, 1060 Vienna, Austria.

[3]Institute of Materials Chemistry, TU Wien, Getreidemarkt 9, 1060 Vienna, Austria.

[†] These two authors contributed equally.

Number of pages: 33

Number of figures: 23

Number of tables: 6

Table of Contents

S.1 Preparation of Silicon Oxycarbide Supports 7a and 7b 3

S.1.1 Powdered Silicon Oxycarbide Supports 7a for Batch Experiments 3

S.1.2 Silicon Oxycarbide Monoliths 7b for Continuous-Flow Experiments 3

S.2 Characterization of Supports and SILPs 5

S.2.1 Nitrogen Physisorption Measurements 5

S.2.2 Permeability Measurement of Silicon Oxycarbide Monoliths 7

S.2.3 Field Emission Gun – Scanning Electron Microscopy (FEG-SEM) 8

S.2.4 Thermogravimetric Analysis of Ionic Liquids and SILPs 9

S.2.5 Fourier Transform Infrared Spectroscopy (FTIR) 10

S.2.6 X-Ray Photoelectron Spectroscopy (XPS) 11

S.3 Determination of NMR Conversions and NMR Yields 16

S.3.1 Limonene Carbonate 15 16

S.3.2 Linseed Oil Carbonates 18 18

S.4 Catalyst Screenings 19

S.4.1 Catalyst Screening - Limonene Carbonate 15 19

S.4.2 Continuous Flow - Limonene Carbonate 15 21

S.4.3 Catalyst Screening - Linseed Oil Carbonate 18 22

S.5 Analysis of Ionic Liquid-Based Catalysts (^{13}C-NMR) 23

S.6 Analysis of Cyclic Carbonates (NMR, IR) 26

S.6.1 Limonene Carbonate 15 26

S.6.2 Linseed Oil Carbonate 18 27

S.7 Materials, Methods, and Typical Procedures 28

S.7.1 Materials and Methods 28

S.7.2 Preparation of Powdered SILPs 1a-12a 31

S.7.3 Preparation of Monolithic SiOC-SILPs 1b-2b 31

S.8 List of Abbreviations 32

S.1 Preparation of Silicon Oxycarbide Supports 7a and 7b

S.1.1 Powdered Silicon Oxycarbide Supports 7a for Batch Experiments

For batch experiments using silicon oxycarbide powder **7a**, a preceramic polymer solution **4** with 30 wt.% functionalized polysiloxane and 70 wt.% *tert*-butyl alcohol **3** was used. The ceramic yield determined after pyrolysis (N=4, heating 1 K min^{-1}, 1 h dwell time) is in reasonable agreement with the residual mass determined during thermogravimetric analysis, as shown in **Figure S1**.

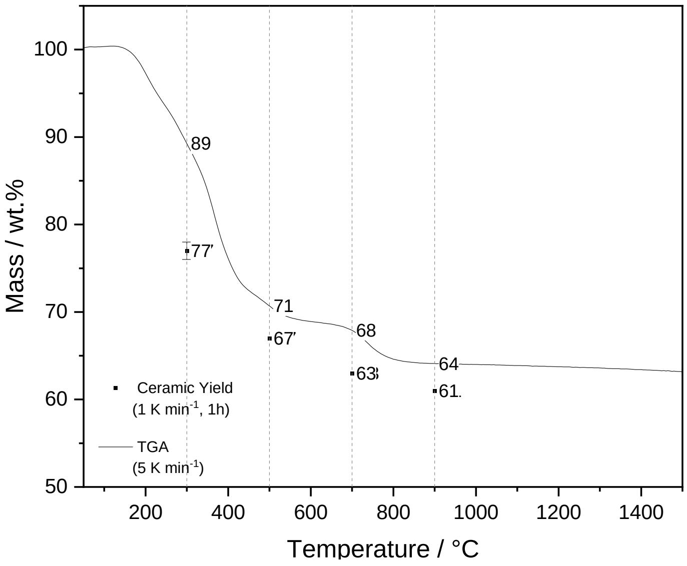

Figure S1: Thermogravimetric analysis in argon flow to study the pyrolytic conversion of the green body **6a** to silicon oxycarbide **7a** and obtained ceramic yields upon pyrolysis at 300, 500, 700, and 900 °C in argon flow.

S.1.2 Silicon Oxycarbide Monoliths 7b for Continuous-Flow Experiments

For continuous experiments using cylindrical, monolithic silicon oxycarbide **7b**, a preceramic polymer solution **4** with 20 wt.% functionalized polysiloxane and 80 wt.% *tert*-butyl alcohol **3** was chosen to combine high porosity and sufficient structural strength. As the pore structure of preliminary samples derived from a preceramic solution **4** using only 10 wt.% functionalized polysiloxane in 90 wt.% *tert*-butyl alcohol **3** collapsed upon freeze-drying and pyrolytic conversion (**Figure S2**), a polymer content of 20 wt.% in solution **4** was selected to result in monoliths of maximum porosity achievable via this method. 20 wt.% polymer content in the solution **4** was chosen for further experiments to combine high porosity and high permeability while providing enough structural strength.

Aluminum molds with an inner diameter of 12 mm were used to generate cylindrical silicon oxycarbide monoliths **7b** with a final diameter of 8.5 mm, thus seamlessly fitting in a column with a 9 mm inner diameter. The linear shrinkage during freeze-drying and pyrolytic conversion was approximately 5 % and 27-29 %, respectively (N=30). The monoliths exhibited a bulk density of 0.4 g cm^{-3} and an apparent porosity of 81 % (water immersion method, N=4).

Cylindrical samples of a length of 6 cm were obtained, a layer of 1.5 mm from the top and bottom part was removed to yield open porosity and parallel bases. The bulk was cut in specimens to stack columns of 220 mm length, requiring a total of 14 specimens. The obtained monolithic specimens had a diameter of 8.4 ± 0.1 mm (N=14).

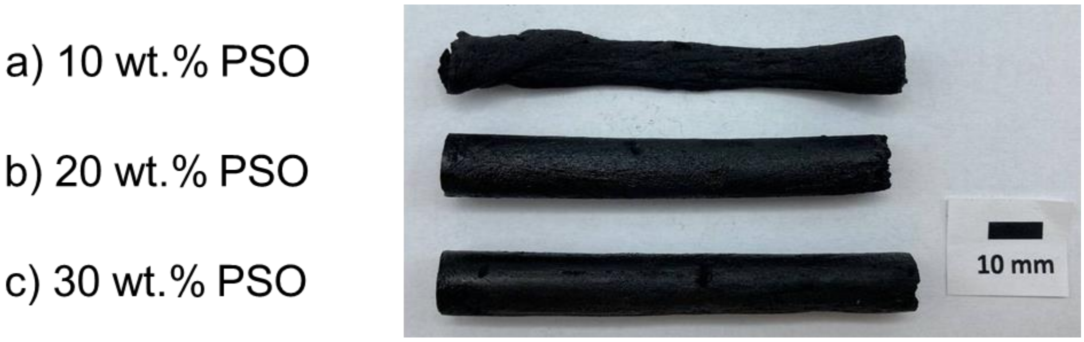

Figure S2: Silicon oxycarbide monoliths **7b** prepared for continuous-flow experiments before cutting 15 mm pieces. Solid loadings of a) 10 b) 20 and c) 30 wt.% polysiloxane **1** in *tert*-butyl alcohol **3** were investigated, whereas b) 20 wt.% were used for all continuous-flow experiments.

S.2 Characterization of Supports and SILPs

S.2.1 Nitrogen Physisorption Measurements

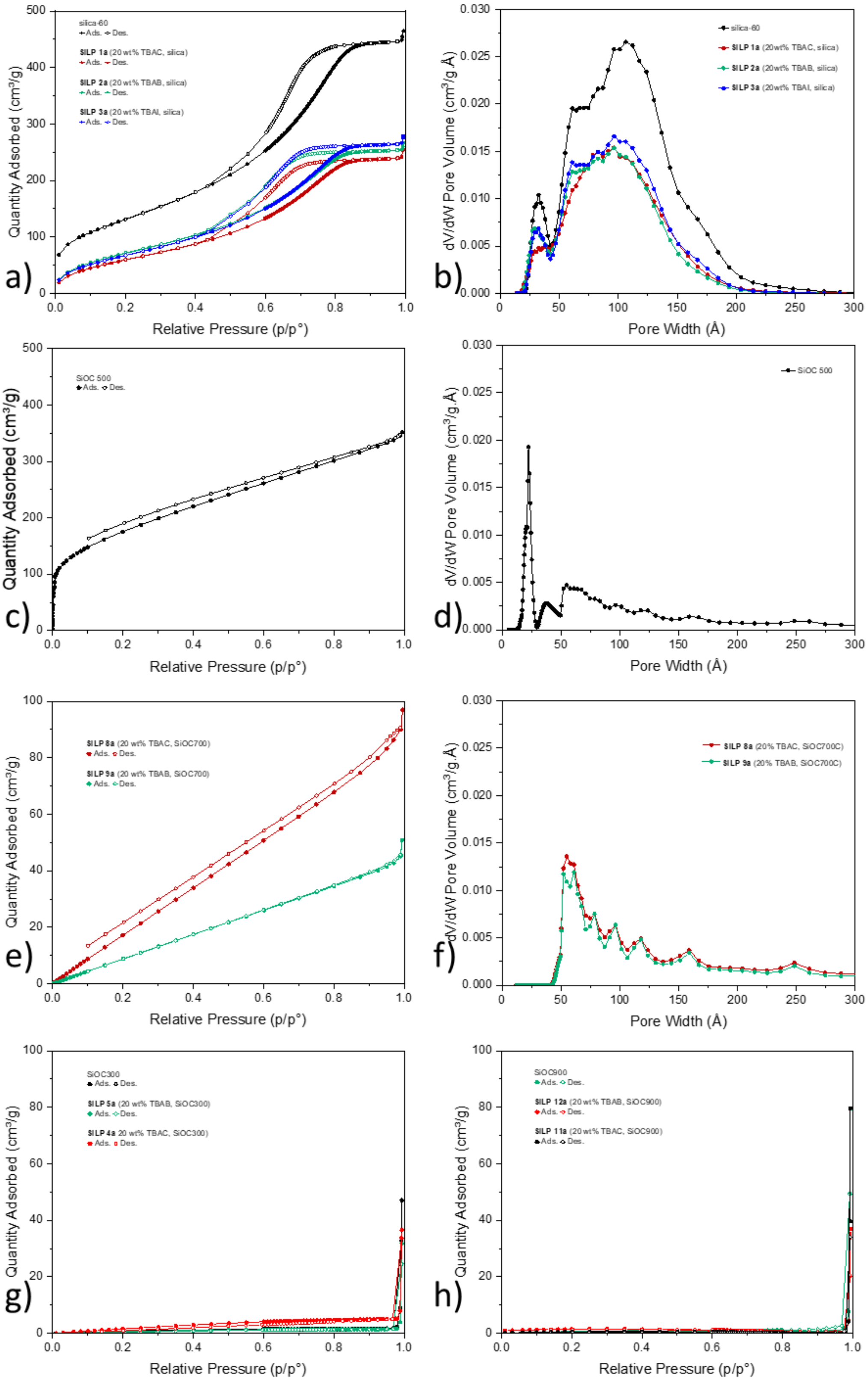

Figure S3: Nitrogen physisorption isotherms (a, c, e, g, h) and pore size distributions (b, d, f) of SILPs and supports.

Table S1: Results from nitrogen physisorption measurements of SiO_2-SILPs and SiOC-SILPs shown in **Figure S3**.

**sample**	**surface area** / $m^2\ g^{-1}$	**pore volume** / $cm^3\ g^{-1}$
SiO_2-60	487.54	0.70
**SILP 1a**	220.88	0.38
**SILP 2a**	262.60	0.39
**SILP 3a**	250.15	0.41
SiOC300	4.67	-
**SILP 4a**	12.58	-
**SILP 5a**	2.56	-
SiOC500	550.39	0.33
**SILP 8a**	143.28	0.15
**SILP 9a**	102.15	0.08
SiOC900	1.95	-
**SILP 11a**	2.25	-
**SILP 12a**	3.84	-

S.2.2 Permeability Measurement of Silicon Oxycarbide Monoliths

Measurements were performed using filtered compressed air as permeating fluid. Shrinking tubes (RS PRO) were used to seal the cylinders in flow direction, 3D printed rings were used to protect sample edges. The permeating gas flow Q was recorded as function of the pressure drop (p_1-p_2). Permeated area A and the sample height L were derived from the sample dimensions. The inlet overpressure p_1 was varied between 0.2 and 2 bar. Permeability constants (Darcian k_1, non-Darcian k_2) were determined using Forchheimer's equation for compressible fluids (**Formula S1**) using least-square fits. The viscosity of air μ was derived from the Sutherland equation (**Formula S2**) and air density ρ was derived from the ideal gas law.[1]

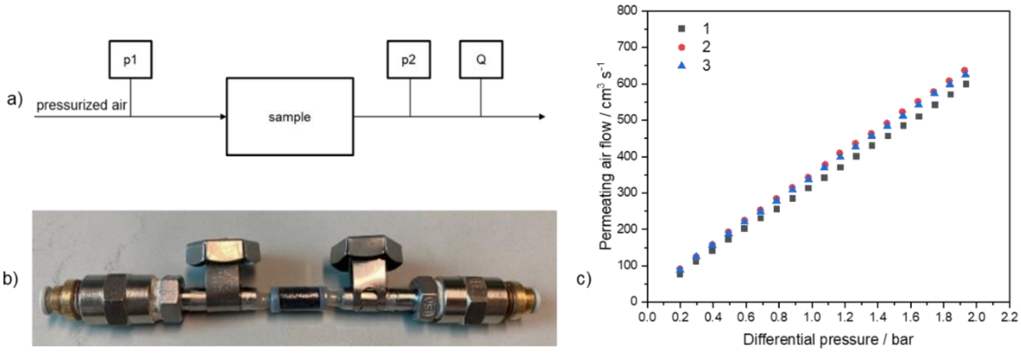

Figure S4: a) Set-up for permeability measurement, b) sample insert using shrinking tubes to seal the sample, and c) measured differential pressure and permeating air flow of SiOC900 monoliths **7b**.

Formula S1: Forchheimer's equation for compressible fluids to determine Darcian (k_1) and non-Darcian (k_2) permeability constants from the measurements shown in **Figure S4 c)**.

$$-\frac{\mathrm{dP}}{\mathrm{dx}} = \frac{p_{in}^2 - p_{out}^2}{2 * p_{out} * L} = \frac{\mu}{k_1}\frac{\mathrm{Q}}{\mathrm{A}} + \frac{\rho}{k_2}\left(\frac{Q}{A}\right)^2$$

Formula S2: Sutherland equation for calculating the viscosity of air used in **Formula S1**.

$$\mu = \mu_0 \left(\frac{T + 273}{273}\right)^{1.5} \left(\frac{273 + C_n}{T + 273 + C_n}\right)$$

dP/dx	pressure gradient along the flow direction
p_{in} , p_{out}	inlet and outlet pressure ($p_{in} = p_{atm} + p_1$; $p_{out} = p_{atm} + p_2$) with p_{atm} from *www.zamg.ac.at*
L	sample length
A	permeating area
ρ	density of the fluid (using ideal gas law and molar mass of air = 29 g mol^{-1})
μ	viscosity of the fluid from Sutherland equation (μ_0 = viscosity of air = 1.73 * 10^{-5} Pa s)
$\frac{Q}{A}$	permeating air flow per permeated area
C_n	Sutherland constant, $C_{n,\,air}$ = 125
T	lab temperature

S.2.3 Field Emission Gun – Scanning Electron Microscopy (FEG-SEM)

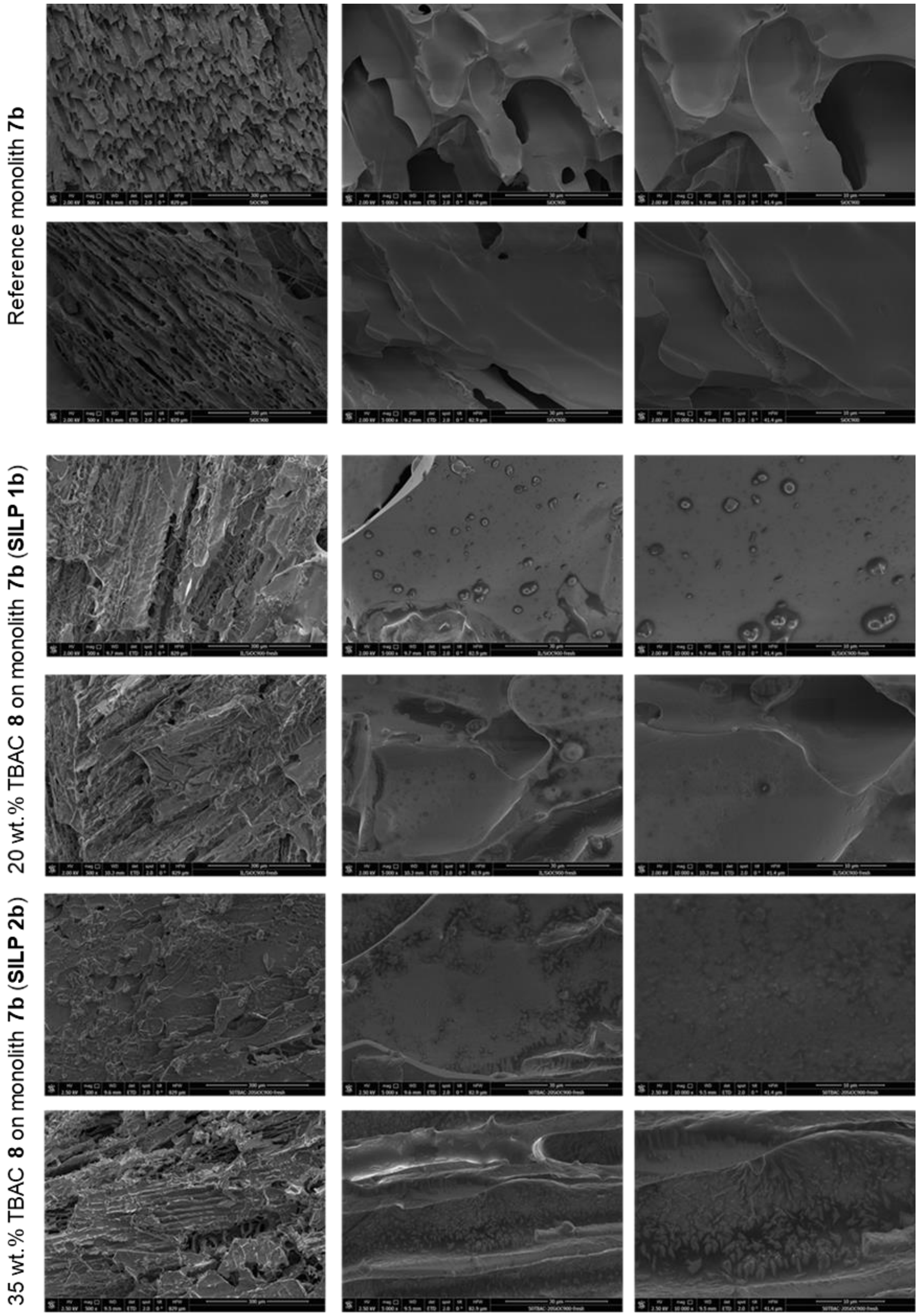

Figure S5: Longitudinal fracture surfaces of monoliths impregnated with different loadings of TBAC **8** showing differences in surface morphology revealed by secondary electron detection in low voltage FEG-SEM as described in ESI chapter S.7.1 .

S.2.4 Thermogravimetric Analysis of Ionic Liquids and SILPs

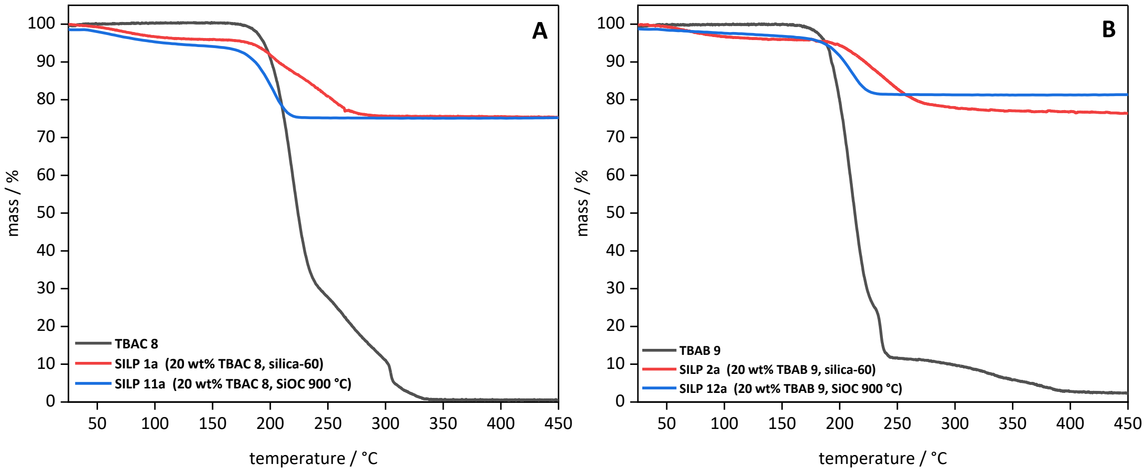

Figure S6: Thermogravimetric analysis in air of ionic liquid, silica- and silicon oxycarbide-based SILPs. (A) TBAC **8** and TBAC-based **SILP 1a** and **SILP 11a**; (B) TBAB **9** and TBAB-based **SILP 2a** and **SILP 12a.** Conditions: 25 °C to 450 °C in air (rate: 5 K min^{-1})

S.2.5 Fourier Transform Infrared Spectroscopy (FTIR)

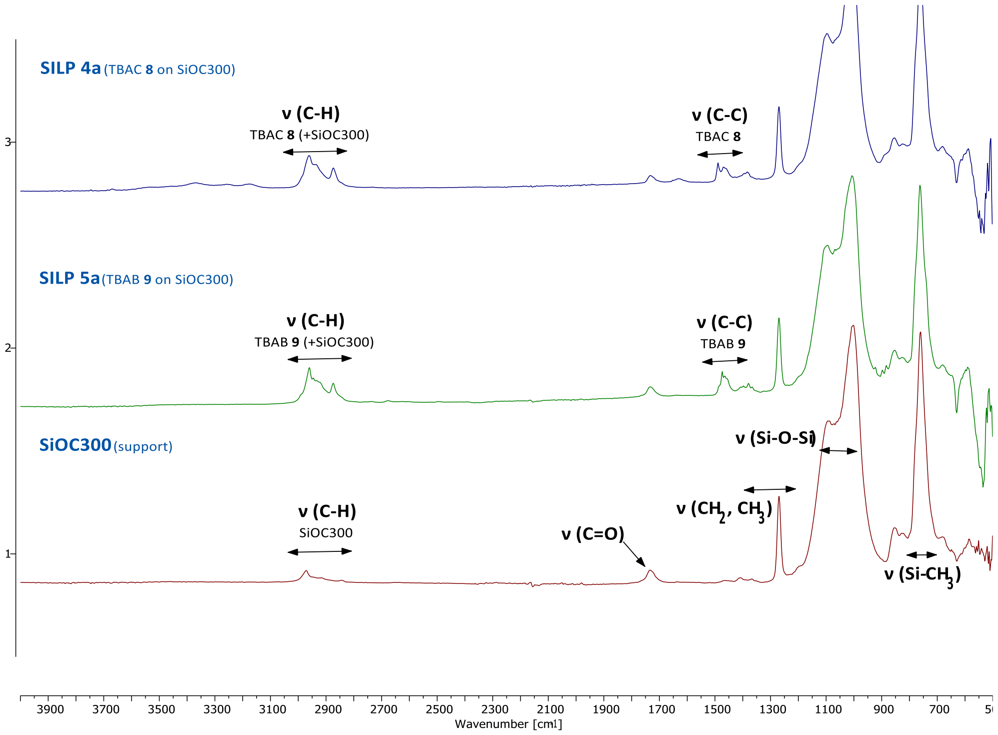

Figure S7: FTIR (ATR) spectrum of **SILP 4a**, **SILP 5a** and SiOC300.

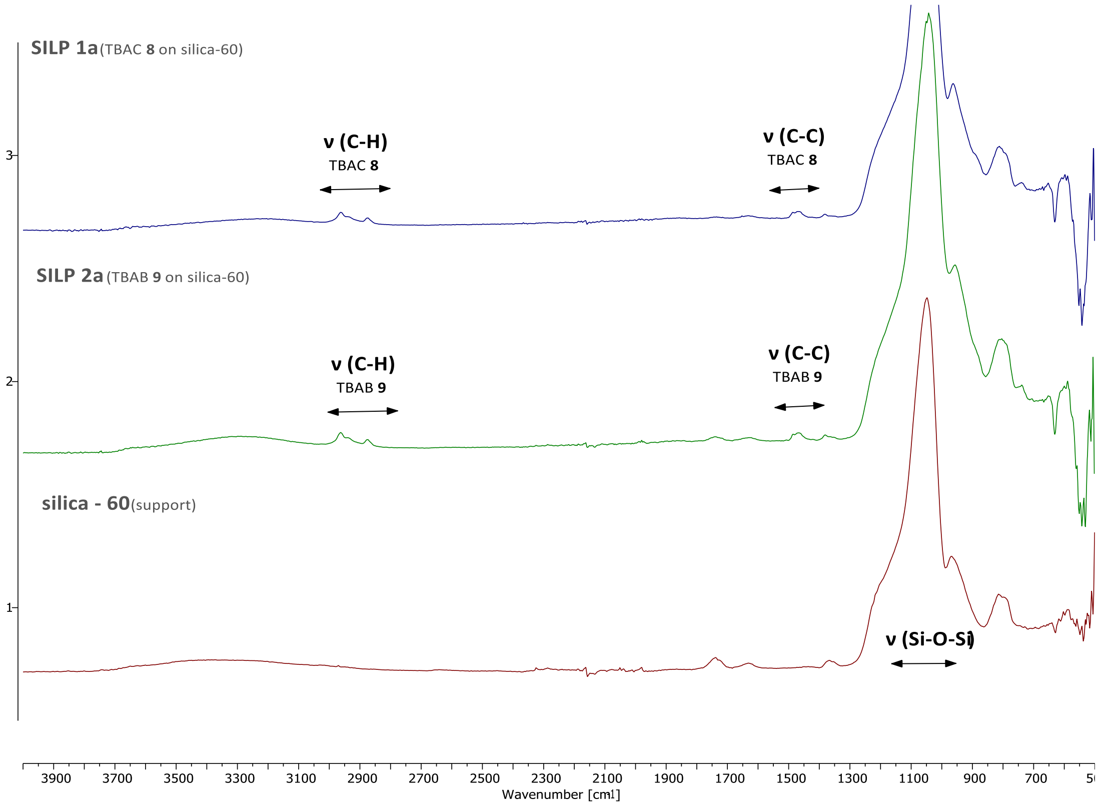

Figure S8: FTIR (ATR) spectrum of **SILP 1a**, **SILP 2a** and silica-60.

S.2.6 X-Ray Photoelectron Spectroscopy (XPS)

All spectra were charge corrected to adventitious carbon at 284.8 eV according to Biesinger *et al.*[2]

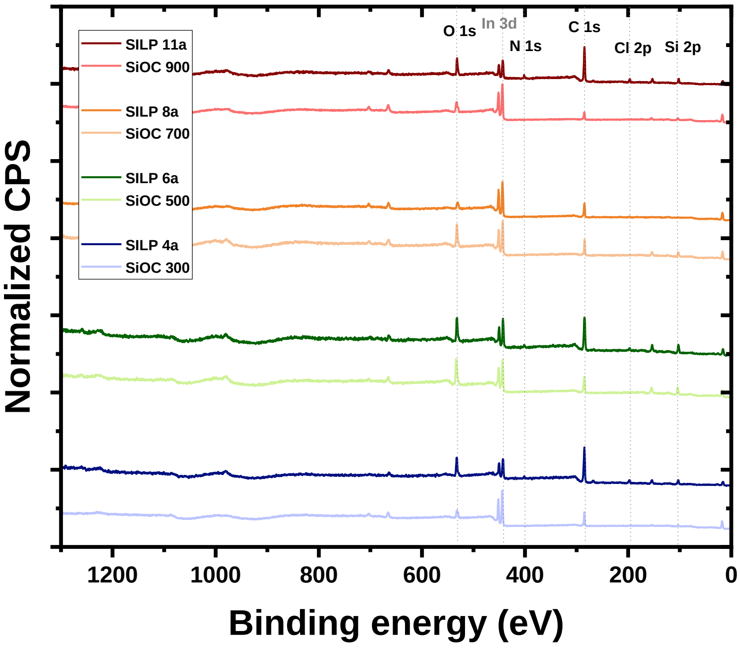

Figure S9: XPS survey of SILPs and supports.

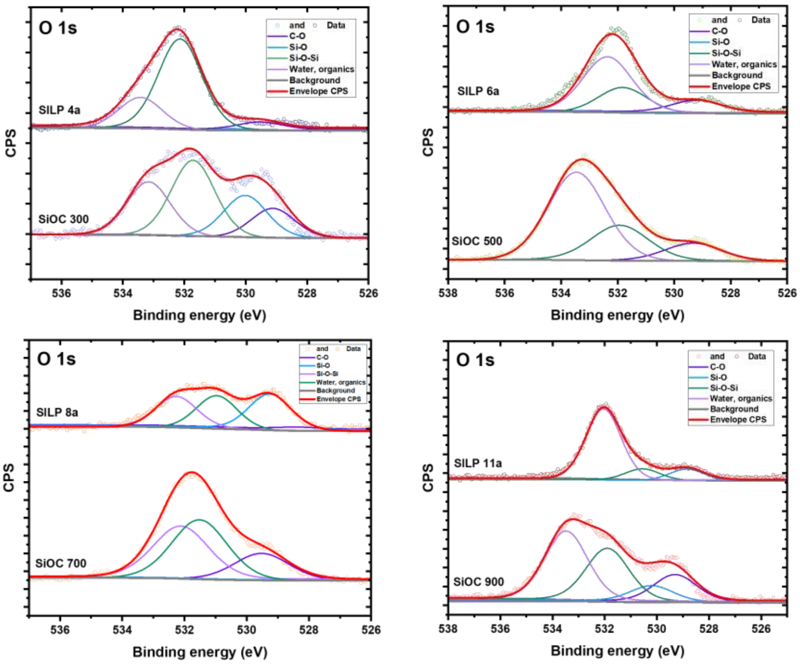

Figure S10: XPS spectra (O 1s) of SILPs and supports.

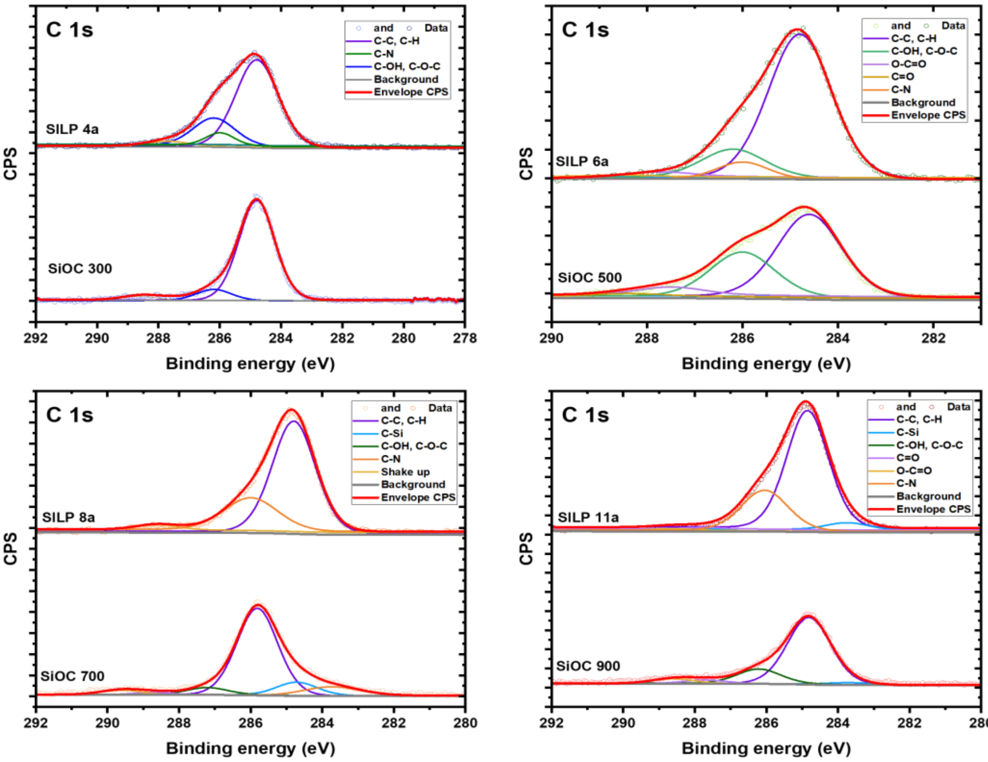

Figure S11: XPS spectra (C 1s) of SILPs and supports.

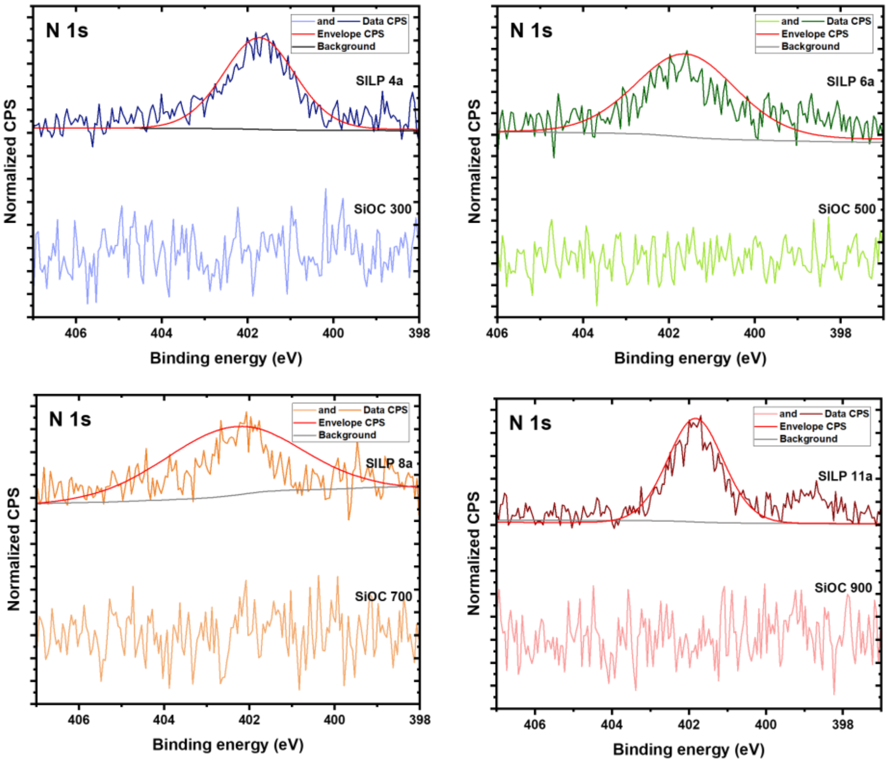

Figure S12: XPS spectra (N 1s) of SILPs and supports.

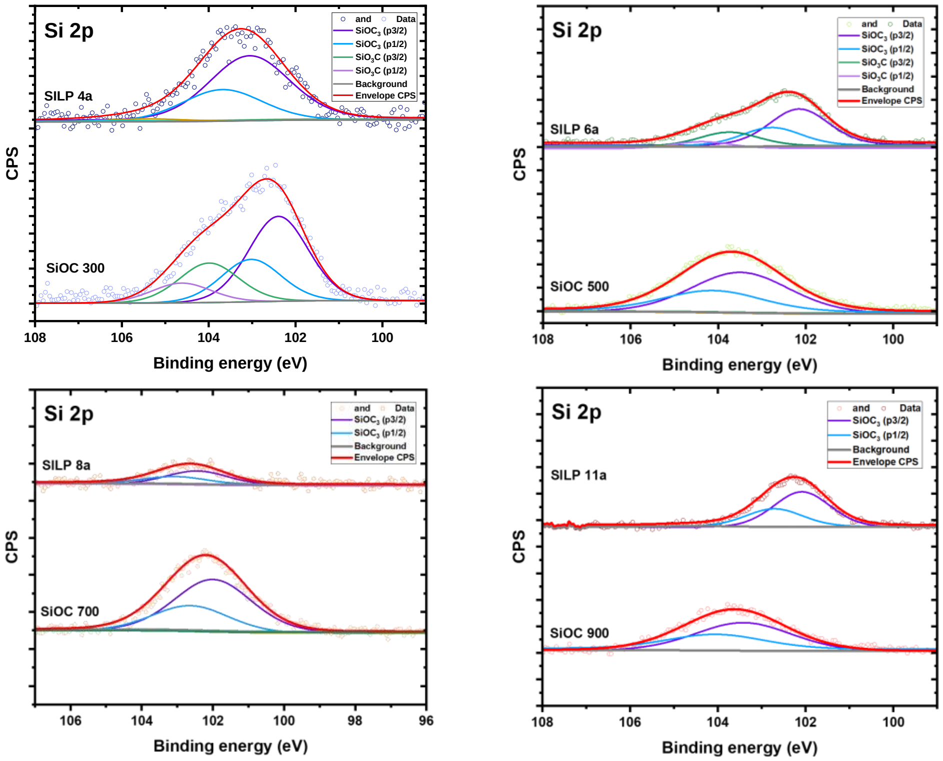

Figure S13: XPS spectra (Si 2p) of SILPs and supports.

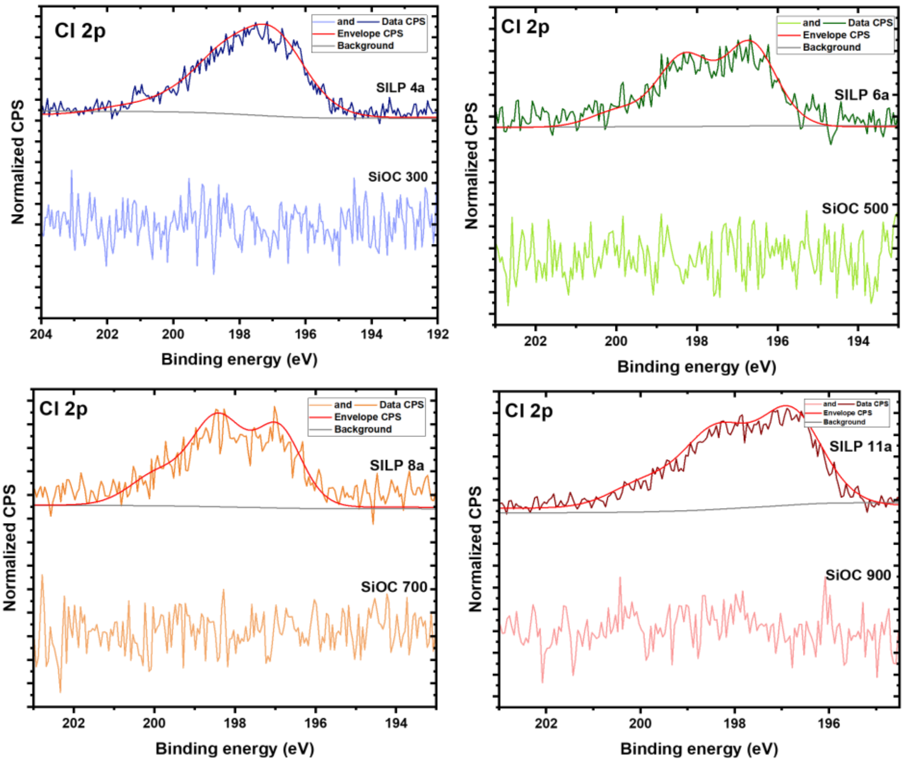

Figure S14: XPS spectra (Cl 2p) of SILPs and supports.

Table S2: Results of quantification of elements in supports and SILPs via XPS.[a]

sample	element	binding energy / eV	concentration / at%
SiOC300	O 1s	531.8	22.6
	C 1s	284.8	67.0
	N 1s	398.3	0
	Si 2p	102.8	10.3
	Cl 2p	202.3	0
**SILP 4a**	O 1s	532.3	14.6
	C 1s	284.8	67.8
	N 1s	401.3	2.0
	Si 2p	102.8	11.8
	Cl 2p	197.8	3.8
SiOC500	O 1s	533.8	30.2
	C 1s	284.8	44.7
	N 1s	410.8	0
	Si 2p	103.8	25.1
	Cl 2p	215.3	0
**SILP 6a**	O 1s	531.8	17.3
	C 1s	284.8	57.5
	N 1s	401.8	3.3
	Si 2p	102.8	19.4
	Cl 2p	197.3	2.6

Continuation of Table S2: Results of quantification of supports and SILPs via XPS. [a]

SiOC700	O 1s	532.3	33.9
	C 1s	284.8	46.9
	N 1s	406.3	0
	Si 2p	102.8	19.2
	Cl 2p	205.3	0
**SILP 8a**	O 1s	530.8	20.3
	C 1s	284.8	71.5
	N 1s	401.8	2.5
	Si 2p	103.3	3.6
	Cl 2p	196.8	2.1
SiOC900	O 1s	532.8	33.5
	C 1s	284.8	48.4
	N 1s	402.8	0
	Si 2p	103.8	18.1
	Cl 2p	198.3	0
**SILP 11a**	O 1s	531.8	12.5
	C 1s	284.8	67.0
	N 1s	401.8	5.3
	Si 2p	101.8	11.3
	Cl 2p	196.8	3.9

[a] Samples were mounted on indium foil and fixed to the stage using double sided carbon tape. Accuracy of XPS measurements falls within 10-20 at% (especially for oxygen and carbon due to adventitious carbon and oxygen) Detection limit in the recorded survey spectra used for quantification lies between 0.1-1 at%.

S.3 Determination of NMR Conversions and NMR Yields

S.3.1 Limonene Carbonate 15

NMR yields and conversions were determined according to a modification of a protocol[3] previously published by our group.

For the batch reactions running for 5 h, NMR spectra at t = 0 h (before reaction) and at t = 5 h (after reaction) were recorded (**Figure S15**). Calculations of yields and conversions were performed according to **Formulas S4-S9** based on integrals of the protons next to the epoxy and carbonate moiety. Naphthalene was used as an internal standard (δ = 7.82 and 7.45 ppm).

Conversions and, therefore, selectivities (given as ratio of yield and conversion) for flow experiments could not be determined due to the volatility of the starting material and, therefore, partial evaporation of the starting material during the release of CO_2 via the back-pressure regulator.

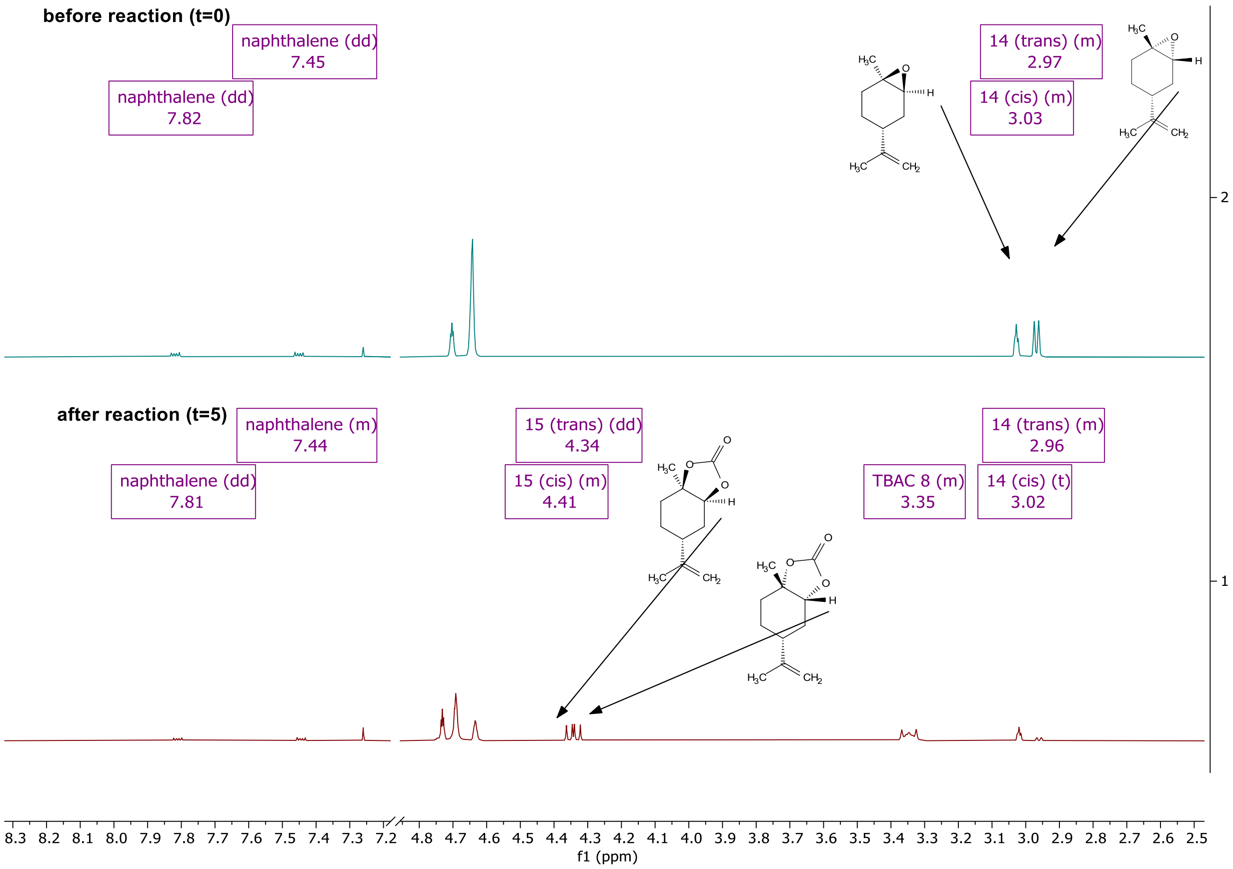

Figure S15: Determination of NMR yields of limonene carbonate **15** via the comparison of the integrals of recorded ^{1}H-NMR spectra at t = 0 h and t = 5 h.

$$\text{conversion } \mathbf{14} \ (cis) \ (\%) = \frac{\mathrm{I}_{11\ (cis)_{\mathrm{t=0}}} - \mathrm{I}_{11\ (cis)_{\mathrm{t=5}}}}{\mathrm{I}_{11\ (cis)_{t=0}}} \cdot 100$$

Formula S4

$$\text{conversion } \mathbf{14} \ (trans) \ (\%) = \frac{\mathrm{I}_{14\ (trans)_{\mathrm{t=0}}} - \mathrm{I}_{14\ (trans)_{\mathrm{t=5}}}}{\mathrm{I}_{14\ (trans)_{t=0}}} \cdot 100$$

Formula S5

$$\text{conversion } \mathbf{14} \ (\%) = \frac{(\mathrm{I}_{14\ (cis)\ \mathrm{t=0}} + \mathrm{I}_{14\ (trans)_{\mathrm{t=0}}}) - (\mathrm{I}_{14\ (cis)_{\mathrm{t=5}}} + \mathrm{I}_{14\ (trans)_{\mathrm{t=5}}})}{\mathrm{I}_{14\ (cis)_{t=0}} + \mathrm{I}_{14\ (trans)_{t=0}}} \cdot 100$$

Formula S6

$$\text{yield } \mathbf{15} \ (cis) \ (\%) = \frac{\mathrm{I}_{15\ (\mathrm{cis})_{\mathrm{t=5}}}}{\mathrm{I}_{14\ (\mathrm{cis})_{t=0}} + \mathrm{I}_{14\ (\mathrm{trans})_{\mathrm{t=0}}}} \cdot 100$$

Formula S7

$$\text{yield } \mathbf{15} \ (trans) \ (\%) = \frac{\mathrm{I}_{15\ (trans)_{\mathrm{t=5}}}}{\mathrm{I}_{14\ (cis)_{t=0}} + \mathrm{I}_{14\ (trans)_{\mathrm{t=0}}}} \cdot 100$$

Formula S8

$$\text{yield } \mathbf{15} \ (\%) = \frac{\mathrm{I}_{15\ (cis)_{\mathrm{t=5}}} + \mathrm{I}_{15\ (trans)_{\mathrm{t=5}}}}{\mathrm{I}_{14\ (cis)_{t=0}} + \mathrm{I}_{14\ (trans)_{\mathrm{t=0}}}} \cdot 100$$

Formula S9

conversion **14**	conversion of *cis* and *trans* limonene oxide **14**
yield **15**	yield of *cis* and *trans* limonene carbonate **15**
$\mathrm{I}_{11\ (\mathrm{xxx})_{\mathrm{t=yy}}}$	integral of *cis* or *trans* limonene oxide **14** at t= 0 h or 5 h
$\mathrm{I}_{12\ (\mathrm{xxx})_{\mathrm{t=5}}}$	integral of *cis* or *trans* limonene carbonate **15** after the reaction (t=5 h)

S.3.2 Linseed Oil Carbonates 18

For the determination of the conversion of epoxidized linseed oil **17**, NMR spectra at t = 0 h (before reaction) and at t = 5 h (after reaction) were recorded (**Figure S16**). Calculation of the conversions was performed according to **Formula S10** based on integrals of the protons next to the epoxy moiety (δ = 3.21 – 2.82 ppm). The signal of the α-CH_2 of the carbonyl group in the backbone of linseed oil, not participating in the reaction, was used as internal standard (δ = 2.29 ppm).

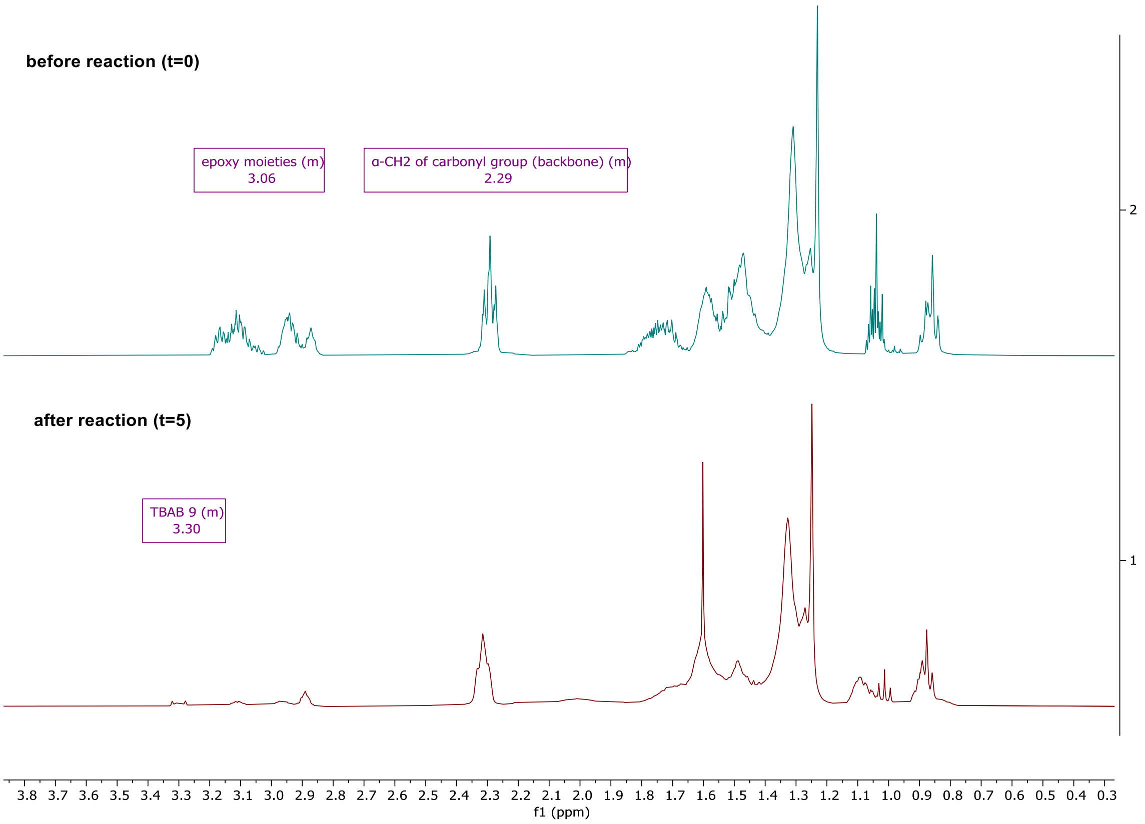

Figure S16: Determination of NMR conversions of epoxidized linseed oil **17** via the comparison of the integrals of recorded ^{1}H-NMR spectra at t = 0 h and t = 5 h.

$$\text{conversion } \mathbf{17}\ (\%) = \frac{I_{17\ t=0} - I_{17\ t=5}}{I_{17\ t=0}} \cdot 100$$

Formula S10

conversion **17**	conversion of epoxidized linseed oil **17**
$I_{17\ t=0}$	integral of epoxy moieties of epoxidized linseed oil **17** at t= 0 h
$I_{17\ t=5}$	integral of epoxy moieties of epoxidized linseed oil **17** at t= 5 h

S.4 Catalyst Screenings

S.4.1 Catalyst Screening - Limonene Carbonate 15

Table S3: Homogeneous catalyst screening for limonene carbonate **15** in batch mode reported in our previous work.[3]

entry	catalyst	conversion (NMR)	yield (NMR)
S1	TBAC **8** (100 °C, 20 h)	72 (*cis*: 43, *trans*: 94)	68 (57[c])
S2	TBAB **9** (100 °C, 20 h)	63 (*cis*: 47, *trans*: 76)	56
S3	TBAI **10** (100 °C, 20 h)	31 (*cis*: 25, *trans*: 35)	12
S4	[C_2mim]Cl **11** (100 °C, 20 h)	18 (*cis*: 17, *trans*: 19)	2
S5	[C_2mim]Br **12** (100 °C, 20 h)	13 (*cis*: 6, *trans*: 17)	6
S6	[C_2mim]I **13** (100 °C, 20 h)	11 (*cis*: 11, *trans*: 11)	0

[a] conditions: 5 mmol limonene oxide **14** (cis/trans = 43/57), 13 mg naphthalene (internal standard), 10 mol % catalyst **8-10**, 5 MPa CO_2 (gaseous, initial pressure), 100 °C, 20 h. [b] conversion of more reactive trans isomer, isolated yield (column chromatography with LP:EA = 10/1– 1/1, 50 g of silica-60)

Table S4: Catalyst screening for the formation of limonene carbonate **15** in batch mode[a]

entry	catalyst	loading	support	conversion[b] (NMR)	yield[b] (NMR)	selectivity[c]
S7	TBAC **8**	-	-	58	55	95
S8	[C_2mim]Cl **11**	-	-	4	0	-
S9	SILP 1a	20 wt% TBAC **8**	SiO_2	55	48	87
S10	SILP 1a (+10 mol% H_2O)	20 wt% TBAC **8**	SiO_2	50	33	66
S11	SILP 2a	20 wt% TBAB **9**	SiO_2	40	25	63
S12	SILP 3a	20 wt% TBAI **10**	SiO_2	66	23	35
S13	SILP 8a	20 wt% TBAC **8**	SiOC700	58	56	97
S14	SILP 9a	20 wt% TBAB **9**	SiOC700	43	43	100
S15	SILP 10a	20 wt% TBAI **10**	SiOC700	8	8	100
S16	SILP 4a	20 wt% TBAC **8**	SiOC300	53	53	100
S17	SILP 6a	20 wt% TBAC **8**	SiOC500	57	56	98
S18	SILP 8a	20 wt% TBAC **8**	SiOC700	58	56	97
S19	SILP 11a	20 wt% TBAC **8**	SiOC900	63	62	98
S20[d]	SILP 11a	20 wt% TBAC **8**	SiOC900	63	61	97
S21	SILP 11a (+10 mol% H_2O)	20 wt% TBAC **8**	SiOC900	62	62	100
S22[e]		-	SiO_2	36	0	-
S23[e]		-	SiOC300	0	0	-
S24[e]		-	SiOC500	4	0	-
S25[e]		-	SiOC700	0	0	-
S26[e]		-	SiOC900	0	0	-

[a] conditions: 0.5 mmol limonene oxide **14** (cis/trans = 43/57), 1.3 mg naphthalene (internal standard), 10 mol% of catalysts **8-10** (homogeneous or physisorbed), 5 MPa CO_2 (gaseous, initial pressure), 120 °C, 5 h; [b] reported as sum of *cis* and *trans* isomers, detailed information about the determination of yield and conversion is summarized in ESI chapter S.3.1 ; [c] ratio of yield (NMR) and conversion (NMR); [d] SILP was prepared using MeOH (instead of CH_2Cl_2) as solvent; [e] 56 mg of supporting material used.

S.4.2 Continuous Flow - Limonene Carbonate 15

Table S5: Continuous production of limonene carbonate **15** with monolithic SiOC-SILPs.

entry	catalyst	loading	flowrate CO_2 [mL/min]	residence time [min]	yield (NMR) [%][c] maximum	yield (NMR) [%][c] overall (12 h /48 h)	leaching[d]
S27[a]	**SILP 1b**	20 wt% PSO<br>20 wt% of TBAC **8**	1.99	7.6	9%	4%	≤ 0.01%
S28[a]	**SILP 1b**	20 wt% PSO<br>20 wt% of TBAC **8**	1.49	10.1	12%	7%	≤ 0.01%
S29[a]	**SILP 1b**	20 wt% PSO<br>20 wt% of TBAC **8**	0.49	30.5	8%	2%	2%
S30[a]	**SILP 2b**	20 wt% PSO<br>35 wt% of TBAC **8**	1.49	10.1	15%	7%	4%
S31[b] (48 h)	**SILP 1b**	20 wt% PSO<br>20 wt% of TBAC **8**	1.49	10.1	9%	7%	≤ 0.01%

[a] conditions: 0.01 mL/min limonene oxide **14** (cis/trans = 43/57), **SILP 1b-2b** (15 – 20 mm monolith pieces, 220 mm in total), 0.49 – 1.99 mL/min CO_2 (15 MPa), 120 °C, 12 h; [b] 48 h experiment (**Figure 9**), other conditions according to footnote a; [c] reported as sum of *cis* and *trans* isomers, detailed information about the determination of yield is summarized in ESI chapter S.3.1 and the experimental part of the main manuscript; Conversions and, therefore, selectivities (ratio of yield and conversion) could not be determined due to partial evaporation of limonene oxide **11** during the release of CO_2 via the back-pressure regulator. [d]limit of detection: 0.1 mg, ≤ 0.1% of total amount of TBAC **8**.

S.4.3 Catalyst Screening - Linseed Oil Carbonate 18

Table S6: Catalyst screening for the formation of linseed oil carbonates **18** in batch mode.[a]

entry	catalyst	loading	support	conversion (NMR)
S32	TBAC **8**		-	41%
S33	TBAB **9**		-	56% (97%)[b]
S34	TBAI **10**		-	46%
S35	[C_2mim]Br **12**		-	43%
S36	**SILP 1a**	20 wt% TBAC **8**	SiO_2	31%
S37	**SILP 2a**	20 wt% TBAB **9**	SiO_2	43%
S38	**SILP 3a**	20 wt% TBAI **10**	SiO_2	48%
S39	**SILP 8a**	20 wt% TBAC **8**	SiOC700	45%
S40	**SILP 9a**	20 wt% TBAB **9**	SiOC700	66%
S41	**SILP 10a**	20 wt% TBAI **10**	SiOC700	67%
S42	**SILP 5a**	20 wt% TBAB **9**	SiOC300	75%
S43	**SILP 7a**	20 wt% TBAB **9**	SiOC500	43%
S44	**SILP 9a**	20 wt% TBAB **9**	SiOC700	66%
S45	**SILP 12a**	20 wt% TBAB **9**	SiOC900	62%
S46		-	SiO_2	2%
S47		-	SiOC300	0%
S48		-	SiOC500	0%
S49		-	SiOC700	0%
S50		-	SiOC900	0%

[a] conditions: 220 mg of **17**, SILP catalyst (0.02 mmol of **8-10**, catalyst loading: 20 wt%), 120 °C, 5 MPa (initial pressure), 5 h. Further details about determination of conversion is given in ESI chapter S.3.2 , [b] reaction time: 20 h.

S.5 Analysis of Ionic Liquid-Based Catalysts (^{13}C-NMR)

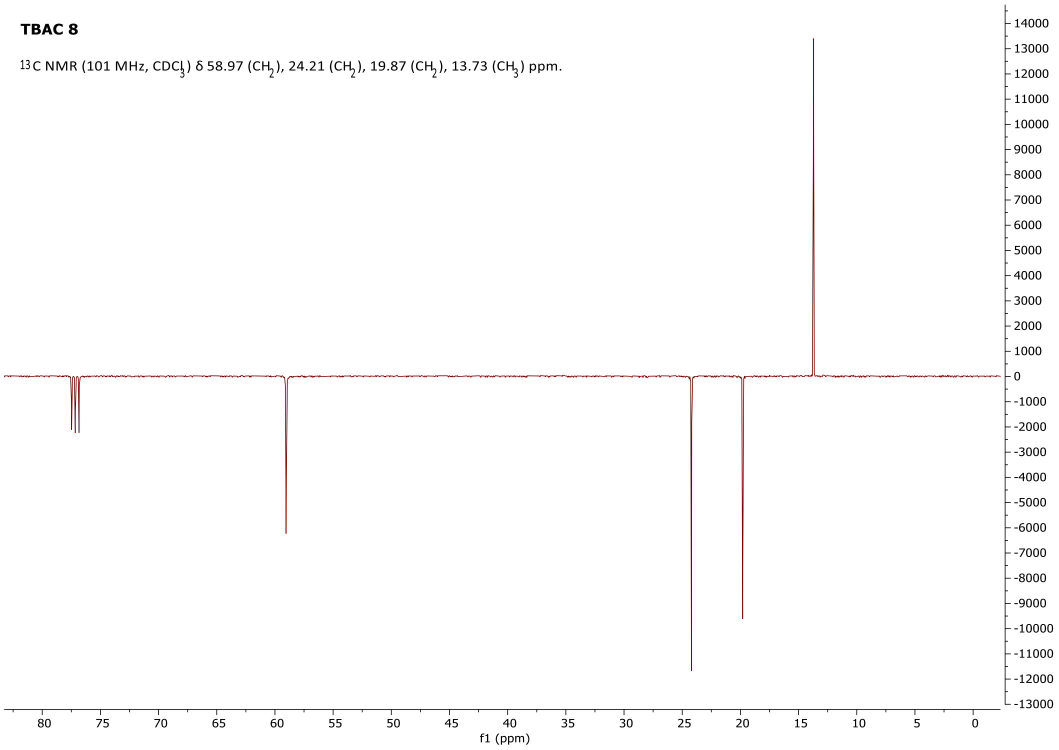

Figure S17: ^{13}C-NMR spectra of tetrabutylammonium chloride TBAC 8.

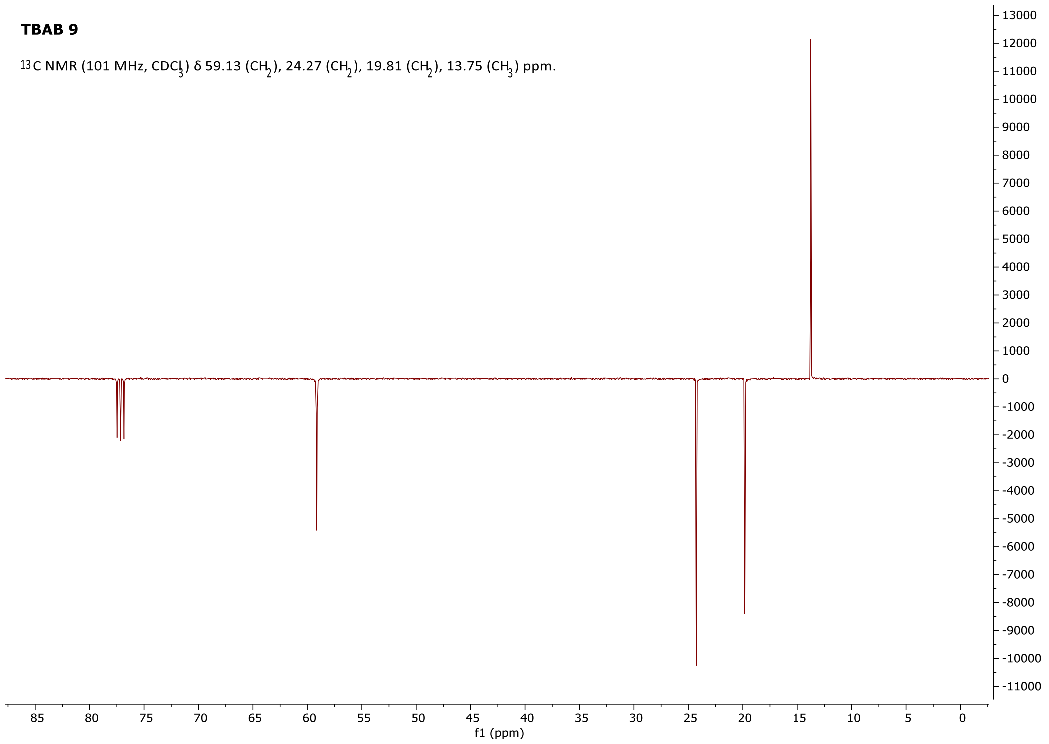

Figure S18: ^{13}C-NMR spectra of tetrabutylammonium bromide TBAB 9.

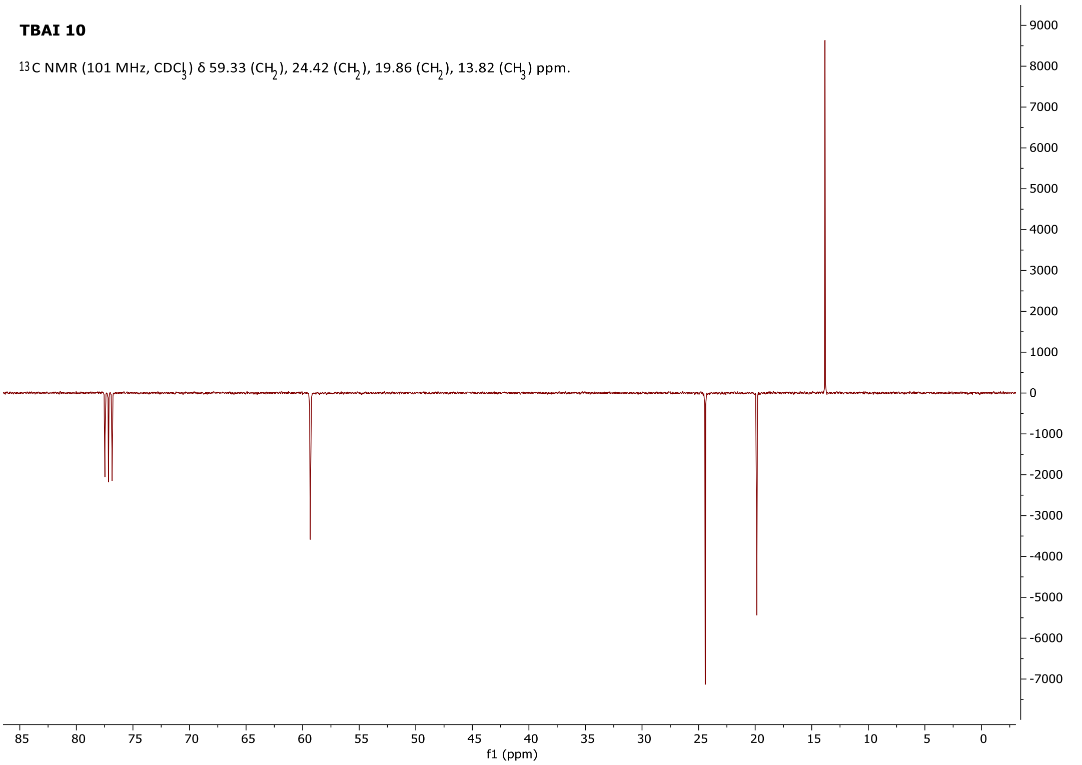

Figure S19: ^{13}C-NMR spectra of tetrabutylammonium iodide TBAI 10.

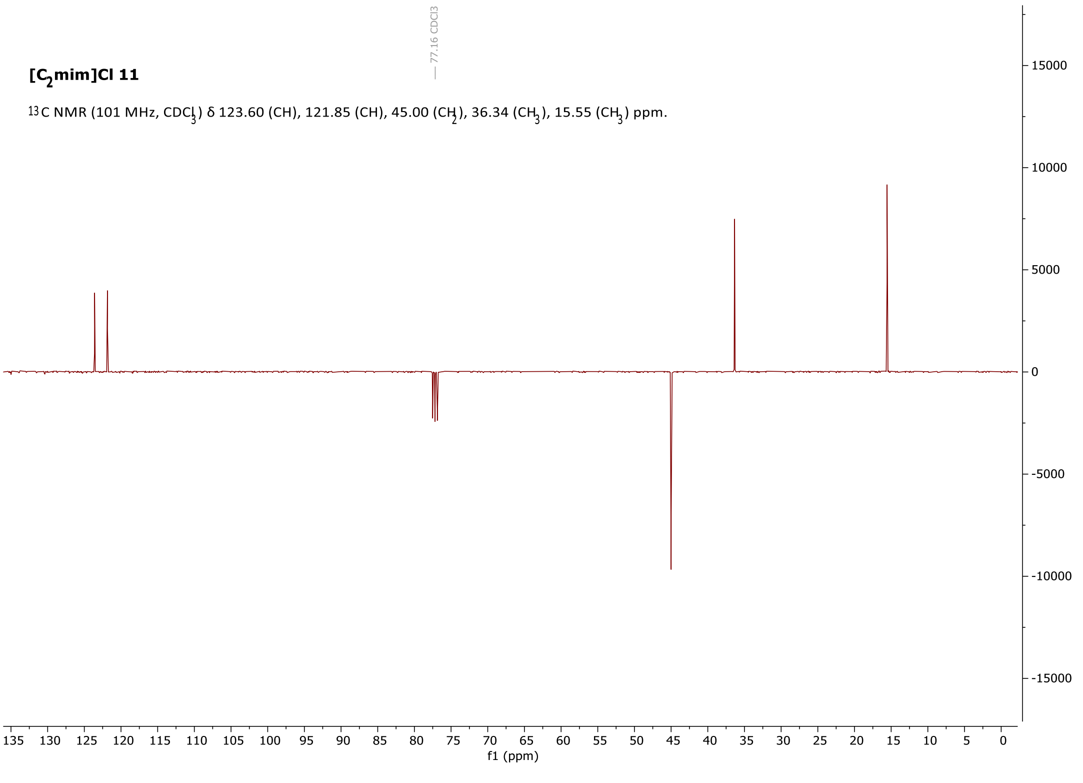

Figure S20: ^{13}C-NMR spectra of 1-ethyl-3-methyl imidazolium chloride [C_2mim]Cl 11.

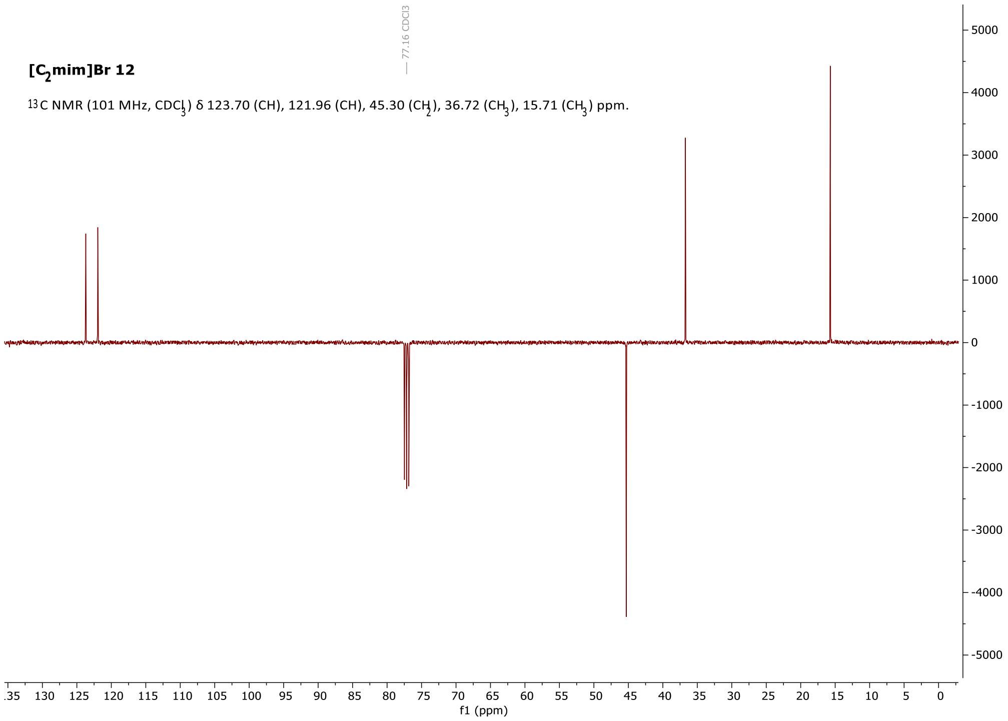

Figure S21: ^{13}C-NMR spectra of 1-ethyl-3-methyl imidazolium bromide [C_2mim]Br 12.

S.6 Analysis of Cyclic Carbonates (NMR, IR)

S.6.1 Limonene Carbonate 15

Analytic data are taken from a previous work[3] published by our group and are reported as a mixture of *cis* and *trans* isomer.

FTIR (ATR, neat): 2942 (alkyl), 1790 (C=O) cm^{-1}

^{1}H-NMR (600 MHz, $CDCl_3$, CH_4Si): δ 4.74 (t, *J* = 1.6 Hz, cis 1H), 4.72 (t, *J* = 1.5 Hz, trans 1H), 4.70−4.68 (m, cis 1H + trans 1H), 4.43−4.40 (m, cis 1H), 4.35 (dd, *J* = 9.5, 7.0 Hz, trans 1H), 2.30−2.18 (m, cis 2H + trans 2H), 2.02−1.94 (m, cis 1H), 1.94−1.85 (m, trans 1H), 1.84−1.74 (m, cis 2H), 1.70 (s, cis 3H), 1.68 (s, trans 3H), 1.67−1.55 (m, cis 1H + trans 2H), 1.49−1.47 (m, cis 3H), 1.45−1.34 (m, trans 5H), 1.24−1.08 (m, cis 1H) ppm.

^{13}C-NMR (101 MHz, $CDCl_3$, CH_4Si): δ 154.87 (trans), 154.61 (cis), 147.53 (cis), 147.42 (trans), 110.27 (trans), 110.05 (cis), 82.78 (cis), 82.24 (trans), 81.93 (cis), 80.66 (trans), 40.01 (trans), 37.42 (cis), 34.26 (cis), 34.07 (trans), 33.14 (trans), 30.66 (cis), 26.36 (cis), 26.27 (trans), 25.77 (trans), 22.35 (cis), 20.99 (cis), 20.66 (trans) ppm.

S.6.2 Linseed Oil Carbonate 18

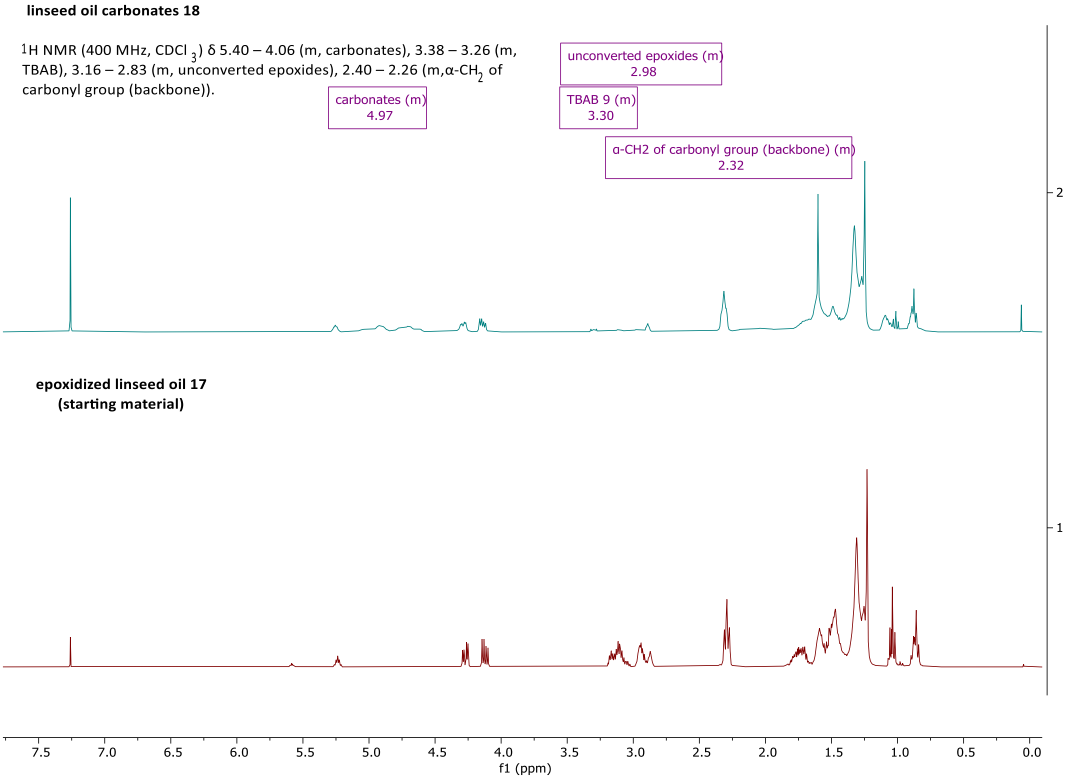

Figure S22: ^{1}H-NMR spectra of linseed oil carbonates 18 and epoxidized linseed oil 17.

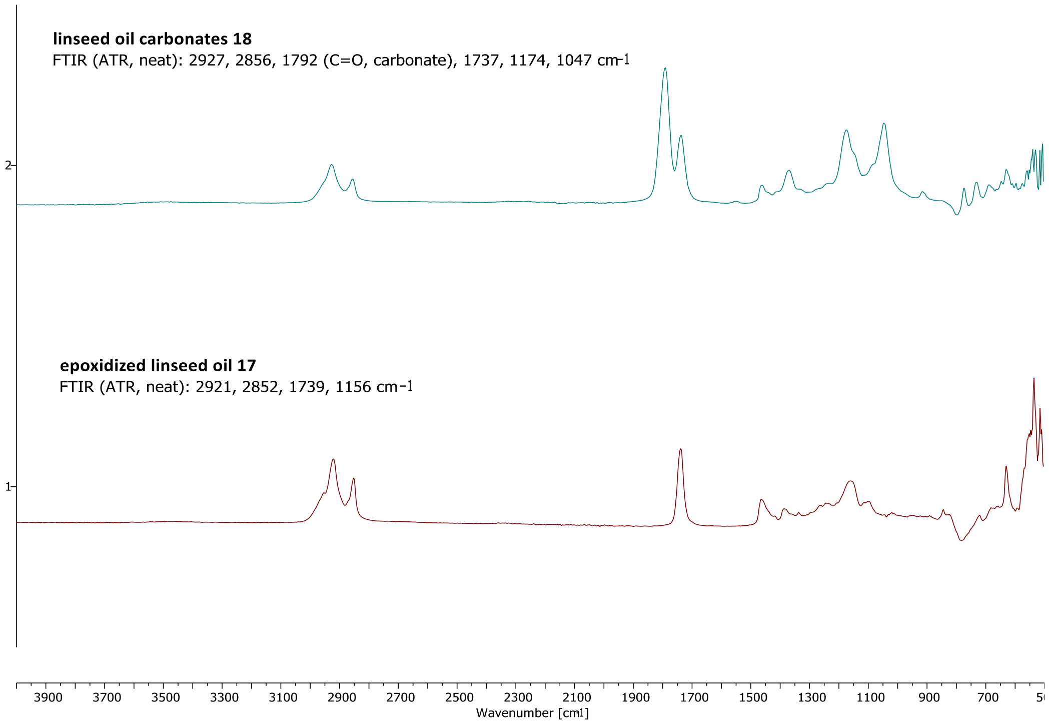

Figure S23: FTIR (ATR) spectra of linseed oil carbonates 18 and epoxidized linseed oil 17.

Spectral data are in accordance with the literature.[4]

S.7 Materials, Methods, and Typical Procedures

S.7.1 Materials and Methods

Chemicals were purchased from several chemical suppliers and used without further purification. Methylsilsesquioxane **1** (Silres, MK) was purchased from Wacker-Chemie. Radical initiator phenyl bis (2,4,6-trimethylbenzoyl) phosphine oxide **5** (Genocure*BAPO) was provided by RAHN. Epoxidized linseed oil **17** (epoxy oxygen content of 8.52 g / 100 g) was provided by HOBUM Oleochemicals. Dichloromethane and methanol were pre-distilled and dried over Al_2O_3 columns (PURESOLV, Innovative Technology).

NMR spectra were recorded from $CDCl_3$ solutions using a Bruker Avance UltraShield 400 spectrometer.

FTIR spectra were recorded on a PerkinElmer Spectrum 65 FTIR spectrometer and the resolution was set to 4 cm^{-1}, 4 scans were used. Spectra were recorded from 4000 - 500 cm^{-1} and raw data were processed with PerkinElmer Spectrum Software.

N_2 physisorption measurements were carried out at a temperature of 77 K using a 3Flex instrument by Micromeritics. Before conducting the measurements, the samples underwent vacuum outgassing at 120 °C for 10 hours. To determine the total pore volume of the samples, the amount of nitrogen adsorbed at $P/P^0 = 0.95$ was evaluated, considering the insignificance of external surface adsorption compared to adsorption within the pores.[5] The apparent surface area was calculated using the Brunauer-Emmet-Teller (BET) equation, following the recommended procedure for microporous sorbents.[6] The relevant pore size distributions were calculated from nonlocal density functional theory (NLDFT) adsorption isotherms considering a cylindrical pore model. The calculations were carried out using the Flex Version 6.01 software provided by Micromeritics Instruments.

Thermogravimetric analysis (TGA) was performed on a Netzsch STA 449 F1 system, and the temperature was gradually increased from 25 °C to 450 °C in air (rate: 5 K min^{-1}). The pyrolytic conversion of polysiloxane **6** (40 mg) to silicon oxycarbide **7** was investigated by TGA (NETZSCH STA 449 C) between 30 and 1500 °C (rate: 5 K min^{-1}) under argon flow (50 mL min^{-1}).

Microscopy was carried out using different microscopes, as follows. A digital microscope (VHX-5000, KEYENCE) was used for optical microscopy. To further investigate the microstructure, ceramographic sections of cylindric monoliths were prepared by embedding the cut monoliths in epoxy resin (EpoFix, Struers) and polished to a 1 µm diamond finish. The embedded and polished structures were observed using a scanning electron microscope (SEM, FEI Quanta 200) using backscattered electron detection. For investigating the distribution of ionic liquid on monolithic silicon oxycarbide, fracture surfaces of monoliths impregnated with 20 wt.% and 35 wt.% TBAC **8** and a reference **7b** were prepared using a razor blade, samples were dried in high vacuum overnight, mounted on graphitic tape, sputtered with Au (AGAR Sputter Coater, 30 sec) and attached to the sample holder with Ag paste to effectively remove surface charges. The fracture surfaces were investigated using a high-resolution electron microscope with a field emission gun electron source (FEG-SEM, FEI Quanta 2050 FEG) using a low acceleration voltage of 2-2.5 kV and secondary electron detection to obtain morphological contrast.

Solvent adsorption procedure is adapted from literature.[7, 8] The samples were dried for 24 h at 110 °C. 0.5 g dry sample was weighed and exposed to saturated atmosphere of H_2O or *n*-heptane in desiccators at 25 °C for 24 h and weighed again. The measurement was conducted in triplets.

Water immersion following DIN EN 623-2[9] for monolithic ceramics was employed to determine bulk density, apparent solid density, and apparent porosity.

Mercury intrusion porosimetry (Pascal 140/440, POROTEC) was used for the determination of pore-opening diameters and pore-size distribution.

X-ray photoelectron spectroscopy (XPS) samples were mounted on highly conductive Indium foil. XPS measurements were conducted on a custom-built SPECS XPS apparatus, featuring a monochromatized Al-Kα X-ray source (µFocus 350) with an excitation energy of 1486.6 eV (beam energy and spot size: 70 W onto 400 µm, angle: 51° to sample surface normal) and a hemispherical WAL-150 analyzer (acceptance angle: 60°). The instrument maintained a base pressure of $5 \cdot 10^{-10}$ mbar, while the pressure during measurements in the analysis chamber was $8 \cdot 10^{-9}$ mbar. Survey spectra were captured at pass energies of 100 eV, while detailed spectra utilized pass energies of 30 eV. Data analysis was performed using CasaXPS software and Scofield sensitivity factors[10]. Transmission corrections, following the instrument vendor's

specifications, were applied. For all elements Shirley backgrounds were employed[11]. Charge correction was implemented using the C 1s peak for adventitious carbon, shifting it to 284.8 eV binding energy (BE) following the methodology outlined by Biesinger *et al*[2]. Accuracy of XPS measurements falls within 10-20% of the values presented (in units of relative atomic percent (at%)), and the detection limit in survey measurements used for quantification ranges from 0.1-1 at%, varying based on the element.

Permeability measurements are described in detail in ESI chapter S.2.2 .

Autoclave experiments were carried out in a pressure vessel from Berghof (BR-40, PTFE insert 40 mL, PTFE sealing (suitable for $scCO_2$), temperature controller: BTC-3000, Manometer LEO3 from Keller).

Continuous-flow experiments were conducted with a $scCO_2$ continuous flow device from Jasco (Jasco Corporation, Tokyo, Japan). Carbon dioxide, purchased from Messer Austria GmbH (> 99.995 % purity; with ascension pipe), was cooled to -7 °C by a recirculating cooler (CF 40, JULABO GmbH) and was introduced by two CO_2-pumps (PU-2086Plus) with cooled heads. An HPLC pump (PU-2089Plus) delivered substrates. Empty 316 stainless steel HPLC columns from DuPont (Zorbax, bio series, GF-250; 250 mm x 9.4 mm ID x 12.7 mm OD, 2 µm frits, 17.35 mL volume) were used as catalyst cartridges. Impregnated silicon oxycarbide monoliths (220 mm; 15-20 mm pieces, 15.27 mL) were loaded into a shrinking tube (RS PRO). The shrunk monoliths were inserted into the catalyst cartridge which was heated up in an HPLC column oven (Brinkmann CH-500 HPLC column heater system, up to 150 °C). Substrates were additionally preheated to 80 °C in a preheating coil (CO-2060Plus). Carbon dioxide was released via a back-pressure regulator (BP-2080Plus, temperature set to 60 °C) and the product was collected in 30 mL glass vials with cyclones (product collector: SCF-Vch-Bp). All parts were connected with 1/16” stainless steel tubing.

S.7.2 Preparation of Powdered SILPs 1a-12a

Silicon oxycarbide cubes **7a** prepared from 30 wt.% preceramic solution **4** were milled (30 s^{-1}, 30 s, ZrO_2 inlet) using a vibrating mill (Retsch MM 40) and sieved (< 90 µm, DIN 4188) to obtain particle sizes comparable to silica-60.

For SILPs,[3] ionic liquid **8-10** (20 wt%, dried under high vacuum for 1 d) was dissolved in dry dichloromethane (silica-60: 100 mL; silicon oxycarbide: 50 mL) and silica-60 (21.000 g, 80 wt%) or silicon oxycarbide (0.800 g, 80 wt%), dried in a vacuum oven (50 °C, 50 mbar, 3 d) was added. The suspension was shaken for 1 h at 480 rpm. Solvent was removed in vacuo and SILP was further dried under high vacuum for 1 d.

S.7.3 Preparation of Monolithic SiOC-SILPs 1b-2b

For continuous-flow experiments, 1.5 mm from the bottom and top part of the cylinders (5-6 cm) was removed and the samples were cut (Struers Minitom, diamond cut-off wheel, 150 rpm) to monolith specimen **7b** of about 15-16 mm. TBAC **8** (for **SILP 1b**: 20 wt%, for **SILP 2b**: 35 wt% dried under high vacuum for 1 d) was dissolved in 100 mL of dry MeOH and monoliths (220 mm as 15 mm pieces, approx. 4.9 g, 80 wt%) were added. The suspension was treated in an ultrasonic bath for 2 h at 40 °C. Solvent was removed under vacuo and monolithic SiOC-**SILP 1b-2b** was further dried under high vacuum for 1 d. Impregnated monoliths were separated from the remaining ionic liquid and the catalyst loading was determined gravimetrically (**SILP 1b**: 20 wt% of TBAC **8**; **SILP 2b**: 35 wt% of TBAC **8**).

S.8 List of Abbreviations

$[C_2mim]Cl$	1-ethyl-3-methyl imidazolium chloride
$[C_2mim]Br$	1-ethyl-3-methyl imidazolium bromide
$[C_2mim]I$	1-ethyl-3-methyl imidazolium iodide
BET	Brunauer-Emmett-Teller
br	broad (NMR)
d	doublet (NMR)
DCM	dichloromethane
dd	doublet of doublets (NMR)
dt	doublet of triplets (NMR)
EtOAc	ethyl acetate
FEG	field emission gun
IR	infrared (spectroscopy)
J	coupling constant (NMR)
LP	light petroleum (boiling point 40 - 60 °C)
m	multiplet (NMR)
MeOH	methanol
NMR	nuclear magnetic resonance
NLDFT	non-local density functional theory
PDC	polymer-derived ceramic
ppm	parts per million
PSO	polysiloxane
s	singlet (NMR)
SEM	scanning electron microscopy
SILP	supported ionic liquid phase
TBAB	tetrabutylammonium bromide
TBAC	tetrabutylammonium chloride
TBAI	tetrabutylammonium iodide
TGA	thermogravimetric analysis
XPS	X-ray photoelectron spectroscopy
wt%	weight percent
δ	chemical shift (NMR)

References

1. Innocentini, M. D. d. M.; Sepulveda, P.; dos Santos Ortega, F., Permeability. In *Cellular Ceramics*, 2005; pp 313-341.

2. Biesinger, M. C., Accessing the robustness of adventitious carbon for charge referencing (correction) purposes in XPS analysis: Insights from a multi-user facility data review. *Applied Surface Science* **2022,** *597*, 153681.

3. Miksovsky, P.; Horn, E. N.; Naghdi, S.; Eder, D.; Schnurch, M.; Bica-Schroder, K., Continuous Formation of Limonene Carbonates in Supercritical Carbon Dioxide. *Org Process Res Dev* **2022,** *26* (10), 2799-2810.

4. Bähr, M.; Mülhaupt, R., Linseed and soybean oil-based polyurethanes prepared via the non-isocyanate route and catalytic carbon dioxide conversion. *Green Chem.* **2012,** *14* (2), 483-489.

5. Aharen, T.; Habib, F.; Korobkov, I.; Burchell, T. J.; Guillet-Nicolas, R.; Kleiz, F.; Murugesu, M., Novel Co-based metal–organic frameworks and their magnetic properties using asymmetrically binding 4-(4′-carboxyphenyl)-1,2,4-triazole. *Dalton Transactions* **2013,** *42* (21), 7795-7802.

6. Sanchez-Varretti, F. O.; Garcia, G. D.; Ramirez-Pastor, A. J.; Roma, F., A simple model for studying multilayer adsorption of noninteracting polyatomic species on homogeneous and heterogeneous surfaces. *J Chem Phys* **2009,** *130* (19), 194711.

7. Prenzel, T.; Guedes, T.; Schlüter, F.; Wilhelm, M.; Rezwan, K., Tailoring surfaces of hybrid ceramics for gas adsorption–from alkanes to CO2. *Separation and Purification Technology* **2014,** *129*, 80-89.

8. Szoldatits, E.; Essmeister, J.; Schachtner, L.; Konegger, T.; Föttinger, K., Polymer-derived SiOC as support material for Ni-based catalysts: CO2 methanation performance and effect of support modification with La2O3. *Frontiers in Chemistry* **2023,** *11*.

9. DIN, Advanced technical ceramics; monolithic ceramics; general and textural properties; part 2: determination of density and porosity; German version EN 623-2:1993. 1993; pp 1-8.

10. Scofield, J. H., Hartree-Slater subshell photoionization cross-sections at 1254 and 1487 eV. *Journal of Electron Spectroscopy and Related Phenomena* **1976,** *8* (2), 129-137.

11. Shirley, D. A., High-Resolution X-Ray Photoemission Spectrum of the Valence Bands of Gold. *Physical Review B* **1972,** *5* (12), 4709-4714.
